# Bayesian localization of CNV candidates in WGS data within minutes

**DOI:** 10.1186/s13015-019-0154-7

**Published:** 2019-09-23

**Authors:** John Wiedenhoeft, Alex Cagan, Rimma Kozhemyakina, Rimma Gulevich, Alexander Schliep

**Affiliations:** 1 Department of Computer Science and Engineering, University of Gothenburg | Chalmers, Rännvägen 6, 412 58 Gothenburg, Sweden; 2 Department of Computer Science, Rutgers University, Piscataway, NJ 08854 USA; 3 Max Planck Institute for Evolutionary Anthropology, 04103 Leipzig, Germany; 4 Wellcome Trust Sanger Institute, Hinxton, CB10 1SA UK; 5 Institute of Cytology and Genetics of the Siberian Branch of the Russian Academy of Sciences, Novosibirsk, 630090 Russia

**Keywords:** HMM, Wavelet, CNV, Bayesian inference

## Abstract

**Background:**

Full Bayesian inference for detecting copy number variants (CNV) from whole-genome sequencing (WGS) data is still largely infeasible due to computational demands. A recently introduced approach to perform Forward–Backward Gibbs sampling using dynamic Haar wavelet compression has alleviated issues of convergence and, to some extent, speed. Yet, the problem remains challenging in practice.

**Results:**

In this paper, we propose an improved algorithmic framework for this approach. We provide new space-efficient data structures to query sufficient statistics in logarithmic time, based on a linear-time, in-place transform of the data, which also improves on the compression ratio. We also propose a new approach to efficiently store and update marginal state counts obtained from the Gibbs sampler.

**Conclusions:**

Using this approach, we discover several CNV candidates in two rat populations divergently selected for tame and aggressive behavior, consistent with earlier results concerning the *domestication syndrome* as well as experimental observations. Computationally, we observe a 29.5-fold decrease in memory, an average 5.8-fold speedup, as well as a 191-fold decrease in minor page faults. We also observe that metrics varied greatly in the old implementation, but not the new one. We conjecture that this is due to the better compression scheme. The fully Bayesian segmentation of the entire WGS data set required 3.5 min and 1.24 GB of memory, and can hence be performed on a commodity laptop.

**Electronic supplementary material:**

The online version of this article (10.1186/s13015-019-0154-7) contains supplementary material, which is available to authorized users.

## Background

Hidden Markov models (HMM) are arguably among the central methods for signal processing. In bioinformatics, they are commonly used for the detection of copy-number variations (CNV), which have been recognized to play an important role in cancer progression [1–3] and neuropsychiatric disorders [4, 5]. Depending on the application and experimental platform, the number of states would be chosen between 3 for simple gains and losses, to around 10 for complex genomic alterations in certain cancers. Since CNV can disrupt or duplicate genes and regulatory elements, effects such as loss-of-function, chimeric proteins, as well as gene dosage can lead to variations in phenotype. Copy-number variants fixed in divergently selected populations can be used as candidates for genetic causes underlying phenotypic adaptations.

The challenges in HMM segmentation of WGS data are two-fold. First, though the advantages of Bayesian segmentation over frequentist approaches have previously been noted [6–10], inference is computationally demanding on WGS-scale data; in particular, Bayesian methods which rely on Markov Chain Monte Carlo (MCMC) approximations are infeasible on standard computers, in terms of memory requirements, speed and convergence characteristics. Second, HMM assume piecewise constant data with variates conditionally independent given the true segmentation, which means that any long-range bias violates the model assumptions. Unfortunately, this is the case when using read-depth data from WGS experiments for CNV estimation. The number of reads mapped to any given position is confounded by amplification bias due to primer affinity and GC content, as well as computational bias incurred during read mapping. This can lead to multiple shifts in segment means, as well as non-linear long-range effects in the signal which would be modeled more accurately as piecewise higher-order polynomials. Removing such effects computationally, e.g. by regression methods such as loess [11], is non-trivial, as it requires the separation of three signals: additive experimental noise, a smooth long-range bias as well as the sequence of true means. In other words, it is hard to differentiate between shifts in signal averages which are due to bias and those that represent actual CN changes.

The contributions of this paper aim to address these issues. On the matter of efficient computation, it was recently shown that Bayesian inference of the hidden state sequence using Forward–Backward Gibbs sampling (FBG) [12] can be made feasible for large data sets by using a dynamic compression scheme based on Haar wavelet regression [6]. In this approach, data is presented to the Gibbs sampler in a compressed form, and the sampler adapts the compression dynamically according to the noise level it obtains in each sampling step. This has led to drastic improvements in speed and convergence behavior of FBG. Conceptually, the approach allows the software to “zoom in” on candidate regions for CNV and concentrate its computational efforts there, while ignoring long diploid segments. While the issue of convergence has been addressed and overall speed has been improved [6], memory usage remains an obstacle when analyzing WGS data. Here, we present a novel algorithmic framework to implement the dynamic wavelet compression approach for HMM inference using FBG. We provide new data structures to efficiently store and update marginal state counts for compression block structures, and to efficiently query sufficient statistics at different wavelet resolution levels. We derive a linear time, in-place algorithm for the data transform required for its construction, based on the *lifting scheme* [13].

On the matter of providing FBG with data that fits its model to a reasonable degree, we noticed that it is common practice to sequence sample and control in a multiplexed fashion, often for cost reasons. Using differential read counts from the same, multiplexed sequencing run, see [14] for instance, cancels out any additive coverage bias. This not only reduces the potential for false CNV calls due to systematic shifts in the data, but also obviously decreases the conditional dependence of the observed variates given the true segmentation labels. Using such data is therefore a more appropriate input to HMM methods. Aside from these general considerations, wavelet compression acts favorably on such data: regression relies on a property of wavelets called *polynomial suppression*. If the underlying signal is a polynomial of a degree up to a certain constant, wavelets are orthogonal to it and hence removed during regression. This yields a separation of signal and noise. Higher-order polynomials due to long-range experimental bias however would incur additional discontinuities in the regression, leading to lower compression ratios, higher memory requirements, and, consequently, longer running times of FBG.

**Fig. 1:**
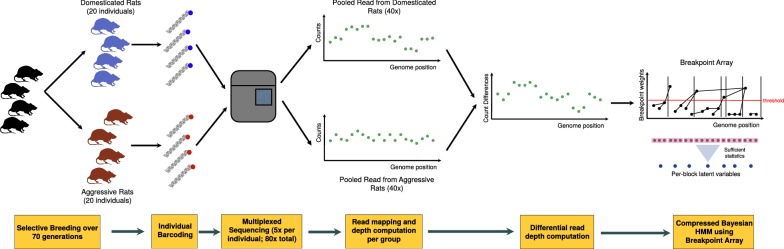
Pipeline for CNV calls in rat populations, divergently selected for tame and aggressive behavior. After individual barcoding and multiplex sequencing, counts of mapped start positions for the tame population are subtracted from those in the aggressive population. This removes shared additive bias from the data. Afterwards, due to low coverage, the data is averaged over 20 positions to make the noise approximately Gaussian. Then, the data is transformed into a breakpoint array data structure, comprised of sufficient statistics as well as a pointer structure to facilitate rapid creation of compressed data blocks depending on a given threshold. The breakpoint array generates block boundaries corresponding to discontinuities obtained in Haar wavelet regression. The universal threshold is used for compression, based on the lowest sampled noise variance in the emission parameters of the HMM during Forward–Backward Gibbs sampling

In order to benchmark our method and demonstrate its applicability to real data, we used it to obtain CNV candidates from differential read depth data for rat populations divergently selected for tame and aggressive behavior (Fig. 1). As expected for a behavioral phenotype, the results are significantly enriched for annotations of neuronal development and function, showing that results are consistent with a hypothesis that CNV play a role in the *domestication syndrome*. To the best of our knowledge, this is the first time fully Bayesian inference on several hundreds of millions of latent state variables has been performed on a commodity laptop within minutes.

**Fig. 2:**
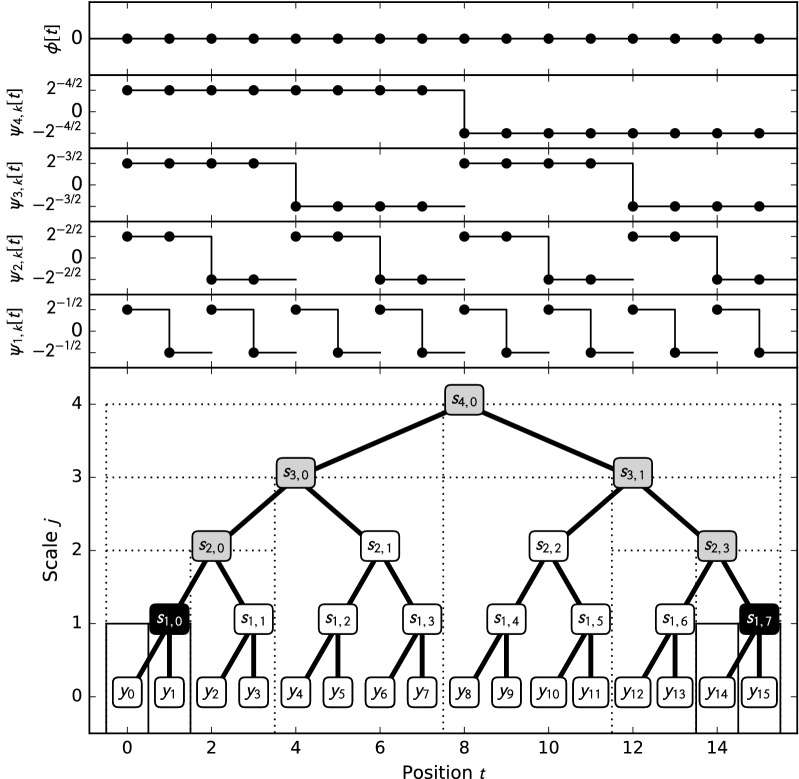
The top subplots show the Haar wavelet basis for $T=16$. The bottom subplot shows the corresponding wavelet tree. In the tree layout induced by the lifting scheme, the position of a coefficient equals that of the central discontinuity of its associated Haar wavelet. For instance, ${\varvec{\psi }} _{2,0}$ has positive support on $\mathbf{y }[0], \mathbf{y }[1]$, and negative support on $\mathbf{y }[2], \mathbf{y }[3]$, with $b^+_{2,0}=0$, $b^\pm _{2,0}=2$ and $b^-_{2,0}=4$. In this example, nodes for which $\left|{} d_{j,k} \right| >\lambda$ are shown in black, i. e. $\left|{} d_{1,0} \right| >\lambda$, inducing block boundaries at 0, 1 and 2, and $\left|{} d_{1,7} \right| >\lambda$, inducing block boundaries at 14, 15 and 16 (indicated by thin solid vertical lines), creating 5 blocks in total. The wavelet tree data structure is subcompressive, as it induces additional breakpoints. $s_{i,k}$ denotes the maximum of all $\left|{} d_{j',k'} \right|$ in the subtree. Nodes in gray indicate the case where $\left|{} d_{j,k} \right| <\lambda$, yet $s_{i,k}>\lambda$, hence inducing additional block boundaries, indicated here by dotted lines, at 2, 4, 8, 12 an 14. This yields a total of 8 blocks

As was shown previously [6, 7], compressing the observed data into blocks of sufficient statistics can significantly speed up Bayesian inference, in particular Forward–Backward Gibbs sampling (FBG). While [7] used a static compression heuristic based on *kd*-trees, we used the discontinuities in the Haar wavelet regression as block boundaries, based on the smallest emission variance among all latent states sampled in each FBG iteration [6]. We used a data structure termed *wavelet tree* to solve the problem of querying sufficient statistics for each block for a given resolution/noise level, without explicitly computing the wavelet regression. We will show that this data structure induces superfluous block boundaries, leading to suboptimal compression ratios, and replace it by a new data structure called a *breakpoint array*. For that, as well as to elucidate the reasoning behind the use of differential read depth data to maximize compression and avoid bias in HMM inference, we briefly review the principles of function regression using wavelet shrinkage: Let $L^2({\mathbb {R}}) :=L^2({\mathbb {R}}, {\mathcal {B}}({\mathbb {R}}), \lambda )$ be the space of square-integrable functions over the reals. This is a Hilbert space with inner product $\left\langle f, g \right\rangle :=\int _{-\infty }^\infty f(x)g(x)dx$. As we are only concerned with functions over subsets of ${\mathbb {R}}$, the inner product commutes without involving the complex conjugate. The inner product induces the norm $\left\| f \right\| :=\sqrt{\left\langle f, f \right\rangle }$. Two functions *f*, *g* are said to be *orthogonal* iff $\left\langle f, g \right\rangle =0$, and a function *f* is called *normal* iff $\left\| f \right\| = 1$. $L^2({\mathbb {R}})$ contains all continuous and piecewise continuous functions, including all piecewise constant functions. Let

$$\psi (t) :={\left\{ \begin{array}{ll} 1 & \quad 0\le t<\frac{1}{2}\\ -1 & \quad \frac{1}{2} \le t < 1\\ 0 & \quad \text {elsewhere} \end{array}\right. }$$

be the Haar wavelet [15], and $\left\{ \psi _{j,k} (t) :=\frac{1}{\sqrt{2^j}} \psi \left( \frac{t- 2^j k}{2^j}\right) \right\}$, ${j, k\in {\mathbb {Z}}}$ (depicted in Fig. 2, top). Since $\left\| \psi _{j,k} \right\| = 1$ and $\left\langle \psi _{j,k}, \psi _{j'k'} \right\rangle = 0$ for $(j,k)\ne (j',k')$, this forms an orthonormal basis of $L^2({\mathbb {R}})$, where a function *y* is represented as the linear combination $y = \sum _{j, k\in {\mathbb {Z}}} \left\langle \psi _{j,k}, y \right\rangle \psi _{j,k}$. The set of detail coefficients $d_{j,k}:=\left\langle \psi _{j,k}, y \right\rangle$ is called the wavelet transform of *y*. A wavelet is said to have *m vanishing moments* if $\left\langle p^i, \psi \right\rangle = 0, 0\le i < m, p \text { constant,}$ it follows that $\psi$ is orthogonal to any polynomial of degree less than *m*, since $\left\langle \sum _{i=1}^{m-1} p^i, \psi \right\rangle = \sum _{i=1}^{m-1}\left\langle p^i, \psi \right\rangle = 0$. This property is called *polynomial suppression* [16]. The Haar wavelet has one vanishing moment, so it is orthogonal only to constant functions.

For computational applications, a vector $\mathbf{f }$ is obtained by sampling *f* at equidistant intervals. The discrete versions of the wavelets are then obtained as ${\varvec{\psi }} _{j,k}[t]:=\psi _{j,k}(t)$ for $t\in {\mathbb {N}}$. These inherit properties such as orthogonality, finite energy and vanishing moments from their continuous counterparts. Let

$$\begin{aligned} b_{j,k}^+ :=2^j k \quad \quad b_{j,k}^\pm :=2^j \left( k+\frac{1}{2}\right) \quad \quad b_{j,k}^- :=2^j (k+1) \end{aligned}$$

be the position after the left, central and right discontinuity of ${\varvec{\psi }} _{j,k}$, respectively.

The Haar wavelet transform is an orthogonal transform, represented by a matrix $\mathcal {W}$ with rows ${\varvec{\psi }} _{j,k}$ Let the observed signal be a sampling of a function *f* corrupted by centered Gaussian noise, i.e. $\mathbf{y } = \mathbf{f } + {\varvec{\epsilon }}, {\varvec{\epsilon }}[t] \sim _{\text {i.i.d.}} N(0, \sigma ^2).$ Since the wavelet transform is linear, it acts on the signal and noise component independently, i.e. $\mathcal {W} \mathbf{y } = \mathcal {W} (\mathbf{f } + {\varvec{\epsilon }}) = \mathcal {W} \mathbf{f } + \mathcal {W} {\varvec{\epsilon }}.$ The central idea in wavelet shrinkage is that $\left\langle \mathbf{f }, {\varvec{\psi }} _{j,k} \right\rangle =0$ if $\mathbf{f }$ is polynomial over the entire support of ${\varvec{\psi }} _{j,k}$ due to polynomial suppression, and, in particular, the support does not span a discontinuity in $\mathbf{f }$. Furthermore, due to orthogonality of $\mathcal {W}$, $\mathcal {W} {\varvec{\epsilon }}$ is again a random vector of i.i.d. random variables distributed as $N(0, \sigma ^2)$, so the noise is maintained under the wavelet transform. In general, orthogonal maps preserve the $L^2$ norm, so $\left\| \mathcal {W} {\varvec{\epsilon }} \right\| = \left\| {\varvec{\epsilon }} \right\|$ and $\left\| \mathcal {W} \mathbf{y } \right\| = \left\| \mathbf{y } \right\|$. It follows that for piecewise polynomial functions with only a few discontinuities, $\left\langle \mathbf{y }, {\varvec{\psi }} _{j,k} \right\rangle = \left\langle {\varvec{\epsilon }}, {\varvec{\psi }} _{j,k} \right\rangle$ for most *j*, *k*, i.e. most wavelet coefficients are only non-zero due to noise. The idea is then to find a way to create a vector $\mathbf{w }$ by setting a suitable set of coefficients in $\mathcal {W} \mathbf{f }$ to zero, and then use the inverse wavelet transform as a regression $\hat{\mathbf{f }} :=\mathcal {W} ^\intercal \mathbf{w }$. The simplest method is to use the *universal threshold* $\lambda _u :=\sqrt{2\ln T}\sigma$ [17], which can be interpreted as the expected maximum deviation of *T* such Gaussian random variables from their mean, as derived by Cramér–Chernoff’s method [18]. Hence, removing coefficients of absolute value below $\lambda _u$ removes all noise coefficients with high probability [17]. Using different variances, the resulting $\hat{\mathbf{f }}$ are piecewise constant functions, whose discontinuities we interpret as block boundaries in a compression scheme. In our approach, $\sigma ^2$ is the minimum variance of all emission parameters in the HMM as sampled at each iteration. The existence of a discontinuity obviously depends on the magnitude of the wavelet coefficients involved: if $\left|{} d_{j,k} \right| >\lambda _u$, then there are block boundaries before data positions $b_{j,k}^+$, $b_{j,k}^\pm$ and $b_{j,k}^-$.

## Implementation

### Block generators

In order to avoid recomputing the wavelet regression explicitly for a new threshold in each FBG iteration, consider the following abstract data structure:

#### **Definition 2.1**

(*Block generator*) Let $\mathbf{b }$ be a vector of breakpoint weights. For a threshold $\lambda$, let $\mathbf{Y }_\lambda$ be a partition of $\mathbf{y }$ into blocks such that there is a block boundary between positions $t-1$ and *t* if $\mathbf{b }[t]\ge \lambda$. We call a data structure a *block generator* if it can, for any threshold $\lambda$, generate an ordered sequence of sufficient statistics that represents $\mathbf{Y }_\lambda$. A block generator is called *compressive* if, for all $\lambda$, $\mathbf{b }[t]<\lambda$ implies that no breakpoint is created between $t-1$ and *t*. It is called *subcompressive* if for some $\lambda$ such a superfluous block boundary is created. A block generator is called *space-efficient* if it stores no more than *T* sufficient statistics, where *T* is the number of input data points.

This definition of a block generator implies that $\mathbf{Y }_{\lambda _1}$ is a subdivision of $\mathbf{Y }_{\lambda _2}$ if $\lambda _1 \le \lambda _2$. For sufficiently small thresholds, we require sufficient statistics for each data point, hence any block generator implementation will have to store a minimum of *T* sufficient statistics. On the other hand, if all entries in $\mathbf{b }$ are unique, each breakpoint subdivides a block defined by a higher threshold, and a simple induction argument shows that a block generator has to be able to generate $2T-1$ different blocks and their sufficient statistics: starting with a single block of size *T* and a sorted sequence of threshold values in $\mathbf{b }$, each threshold creates two new blocks by subdividing one block in the previous partition.

We previously defined the *wavelet tree* data structure to serve as a block generator; for details, see [6]. It is based on the observation that the non-zero support intervals of wavelet basis functions are nested along scales (cf. Fig. 2). Each node corresponds to a basis function, with its position corresponding to the position of the wavelet’s central discontinuity. The wavelet tree stores the maximum absolute coefficient $s_{ij}$ of its subtree in the node. To obtain the sufficient statistics for a block at a given noise level, the tree is traversed in DFS order. Whenever a node is encountered for which $s_{ij}<\lambda$, none of its descendants can have a higher value, and hence no additional discontinuities. The subtree is pruned from the DFS, creating a single block for the sufficient statistics of its leaf nodes. On the other hand, if $s_{ij} \ge \lambda$, the search recurses on the subtrees, creating additional block boundaries between leaves.

Unfortunately, the wavelet tree is subcompressive, as demonstrated by the counterexample in Fig. 2, as well as memory-inefficient, since it stores $2T-1$ statistics. It should be noted that, while the wavelet tree stores as many sufficient statistics as needed for *T* data points, the fact that it is subcompressive implies that the block structures it creates differ from those of a compressive block generator, and hence these are *not* the same $2T-1$ statistics that would occur in across all block structures a compressive block generator would yield.

In order to provide an efficient implementation, we separate a block generator into two sub-structures: a *breakpoint array* to derive a sequence of start and end positions for blocks, and an *integral array* to query the sufficient statistics for each block.

#### Integral array for block statistics

Let a data structure $D(\mathbf{y })$ support the following query: given a start index *s* and an end index *e*, with $s<e$, return the sufficient statistics in the half-open interval [*s*, *e*), i. e. $\sum _{i=s}^{e-1}\mathbf{T }(\mathbf{y }[i])$. A trivial implementation of such a data structure would be to store the statistics of each input position, and then iterate through the array and calculate their cumulative sums between breakpoints. This is obviously costly for huge data, as it incurs $\Theta (N)$ time complexity for a block of size *N*. Constant-time queries could be made by pre-computing all $T^2$ statistics, which is obviously prohibitive for large data.

The basic idea for querying sufficient statistics comes from a simple data structure in image processing called a *summed-area table* or *integral image* [19], which is used to query the sum of a rectangular region in constant time. As its one-dimensional equivalent, let $\mathbf{v }$ be an *integral array* such that

$$\mathbf{v }[t]={\left\{ \begin{array}{ll} \mathbf{T }(0)& \quad t=0\\ \sum _{i=0}^{t-1} \mathbf{T }(\mathbf{y }[t]) & \quad t>0. \end{array}\right. }$$

For any arbitrary start and end positions *s*, *e*, the sufficient statistics of the block [*s*, *e*) can be calculated in constant time as

$$\begin{aligned} \sum _{t=s}^{e-1} \mathbf{T }(\mathbf{y }[t]) = \left( \sum _{t=0}^{s-1} \mathbf{T }(\mathbf{y }[t])\right) - \left( \sum _{i=0}^{e-1} \mathbf{T }(\mathbf{y }[t])\right) = \mathbf{v }[e] - \mathbf{v }[s]. \end{aligned}$$

In contrast to image processing, where integral arrays are constructed over integer data, sufficient statistics require floating-point values for most distributions. Unfortunately, this incurs numeric problems for large data sizes. An IEEE 754 single-precision float has between 6 and 9 significant digits. Assuming that values for sufficient statistics are on the order of 1, the further back a data point is in $\mathbf{v }$, the more of its significant digits is used to store the sum. Neighboring entries will be similar or even equal, leading to catastrophic cancellation for short segments. For instance, values above $\sim$17 million are rounded to multiples of 2, so that even if each entry was 1.0, blocks of size 1 would be queried as 0.

**Fig. 3:**
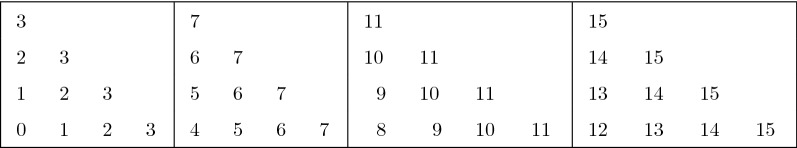
An illustration of an integral array $\mathbf{v }$, using cell size $c=4$. Columns represent data positions, and contain all positions *i* which are added up and stored at $\mathbf{v }[t]$; for instance, $\mathbf{v }[9] = \sum _{i=9}^{11} \mathbf{T }(\mathbf{y }[i])$. The statistics of a block [*s*, *e*) are obtained by adding *v*[*s*], $\mathbf{v }[m]$ for all $s<m<e$, $m\equiv 0\mod c$, and subtracting $\mathbf{v }[e]$ iff $e\not \equiv 0\mod c$. For instance, block [3, 10) is obtained as $\mathbf{v }[3]+\mathbf{v }[4]+ \mathbf{v }[8]-\mathbf{v }[10]$, yielding $\sum _{t=3}^9 \mathbf{T }(y[t])$

To alleviate this, we subdivide $\mathbf{v }$ into non-overlapping *cells* of size *c*, and compute partial cumulative sums of sufficient statistics within each cell; for convenience, we compute these sums from high to low indices, see Fig. 3. It is then easy to see that $\sum _{t=s}^{e-1} \mathbf{T }(\mathbf{y }[t]) = \left( \sum _j \mathbf{v }[j]\right) - \mathbf{v }[e]$ for $j\in \left\{ s\right\} \cup \left\{ i\,\big |\, s<i\le e, i\equiv 0\,\, (\text {mod } c)\right\}$. In our implementation, we used $c=2^{16}=65,\!536$.

#### Breakpoint array for block boundaries

In order to create a block generator, the integral array has to be supplemented with a data structure which yields start and end positions $s_k(\lambda )$, $e_k(\lambda )$ for subsequent blocks *k*. Since $e_k(\lambda )=s_{k+1}(\lambda )$, it suffices to implement an iterator over $s_k$ for increasing *k*, where $s_0 = 0$ and $s_k = e_k(\lambda ) = s_{k+1}(\lambda )$. We use a simple array of pointers to facilitate these queries:

##### **Definition 2.2**

(*Breakpoint array*) Let $\mathbf{b } \in {\mathbb {R}} ^T$ be a vector of breakpoint weights, and $\mathbf{p } \in {\mathbb {Z}} ^T_+$ be a vector of pointers. A data structure $(\mathbf{b }, \mathbf{p })$ is called a *breakpoint array* of input data $\mathbf{y }$ if and only if $\forall t<i<t+\mathbf{p } [t]: \mathbf{b } [t] >\mathbf{b } [i]$. We call each interval $[t, \dots , \mathbf{p } [t]-1]$ a *stretch at t*. A breakpoint array is called *maximal* if for all *T* there exist no $n > \mathbf{p } [t]$ such that setting $\mathbf{p } [t]$ to *n* would still result in a valid breakpoint array.

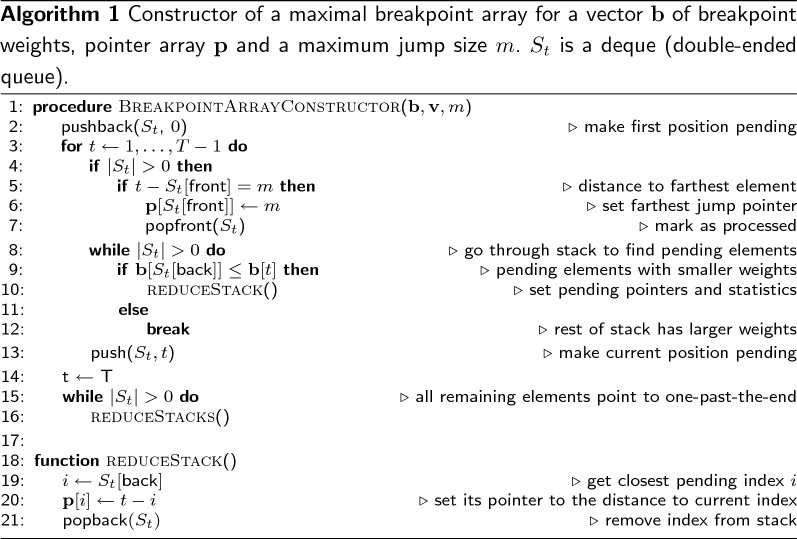



A breakpoint array can be constructed in linear time *O*(*T*) (Algorithm 1), based on a linear-time algorithm to calculate the pointers to the next element at least as large as the current one, which is well established in algorithmic folklore. It is modified here to use the distance to that element instead of a direct pointer (line 20, which would normally read $\mathbf{p }[i] \leftarrow t$). The stack is changed to a deque to accommodate the inclusion of a maximum jump size *m*. The front of the deque is popped and its pointer set whenever it is *m* positions away, which happens at most *T* times.

**Fig. 4:**
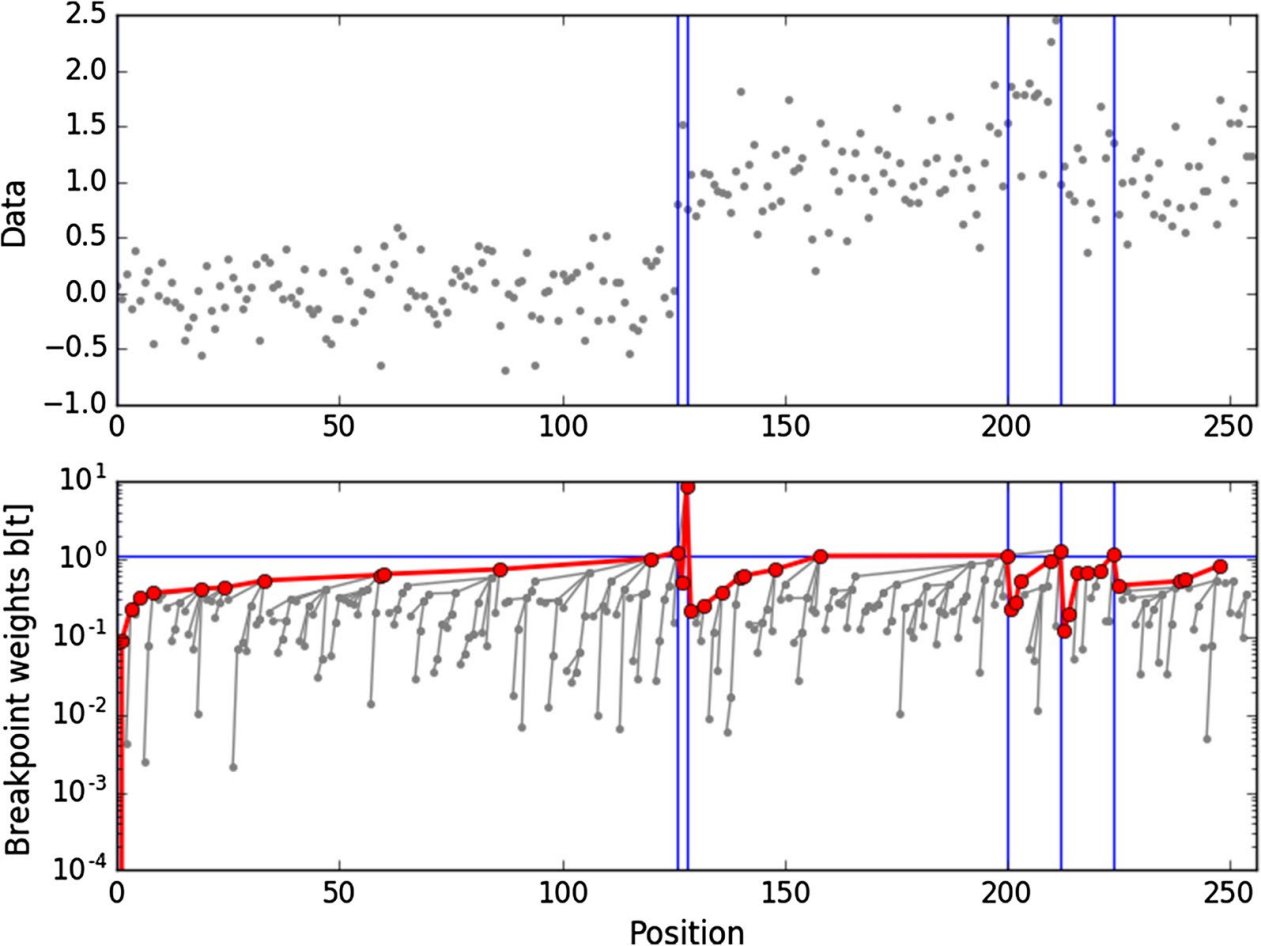
An example of generating blocks following pointers in a breakpoint array. The top figure represents the input data $\mathbf{y }$, the bottom figure represents the absolute wavelet coefficients, as well as the pointers (grey lines) and the path taken by the query (red). Whenever a value above the threshold (horizontal blue line) is found, a breakpoint is returned (vertical blue lines)

For each *t*, $\mathbf{p } [t]$ points to the beginning of next stretch. Within each stretch, the highest breakpoint weight is located at its first position; when searching for weights below a given threshold $\lambda$, once the first weight is found to be below $\lambda$, all others can be safely ignored, leading to a simple query: Starting at $e_{k}(\lambda )+1$, follow pointers until a weight above threshold is encountered (see Fig. 4). In order to derive complexity results, we require the following result:

##### **Theorem 2.1**

(Left-to-right maxima [20, 21]) *For a vector* $\mathbf{x },$ *let* $\mathbf{x }[t]$ *be called a* *left-to-right maximum* *of* $\mathbf{x }$ *iff* $\forall i<t: \mathbf{x }[i]<\mathbf{x }[t].$ *Let* $m_{\mathbf{x }}$ *count the number of left-to-right maximal elements in* $\mathbf{x }.$ *For a random permutation of* $\mathbf{x }$ *with* $\left|{} \mathbf{x } \right| =N$ *elements,* ${\mathbb {E}}\left[ m_{\mathbf{x }}\right] = \sum _{i=1}^N\frac{1}{N} \rightarrow \ln N$ *as* $N\rightarrow \infty.$ *Due to symmetry, the same result holds for minima and right-to-left extrema.*

Following pointers in $\mathbf{p }$ creates a sequence of left-to-right maxima. For a block of size *N*, starting at $e_k(\lambda )$, there are $M:=N-2$ elements in $I:=[e_k(\lambda )+1, \dots , e_k(\lambda )+N=e_{k+1}(\lambda ))$ which can appear in any order, which implies that $e_{k+1}(\lambda )$ can be found in $O(\log N)$ expected time. Likewise, the maximum expected stack size in the constructor (Algorithm 1) is $\ln T$: assume $m=\infty$. An element at *t* is pushed whenever there exists an index *j* on the stack such that $\forall i=j, \dots , \text {top}: \mathbf{w }[i] < \mathbf{w }[t]$. Given the smallest such *j*, the stacks are popped until $\text {top} = j-1$, and $\mathbf{w }[j-1] > \mathbf{w }[t]$. Therefore, the stack contains the right-to-left minima of $\mathbf{w }[1:t]$ after pushing index *t*, and the claim follows from Theorem 2.1 for $t=T$. For any $m<\infty$, the front of the deque gets popped, thus only decreasing the stack size. For the size $T_{hg}$ of the human genome (3.5 billion), the expected maximum stack size is $<22$, a negligible overhead. We noticed that, for noisy data, most entries in $\mathbf{p }$ are much smaller than *T*, and using pointer-sized integers such as size_t in C++ (typically 8 byte on 64-bit systems), would be wasteful. Instead, we use a 2-byte unsigned integer type to accommodate jumps up to $m=65, 536$. The resulting breakpoint array is not maximal anymore, but maintains its space-efficiency and compressivity. The query overhead is minimal in practice; even in case of a single block for genome sized data, $\frac{T_{hg}}{65, 536} < 54$.

#### Haar breakpoint weights

Having established a data structure to iterate over blocks for any given compression level, we now define a vector $\mathbf{b }_H$ of breakpoint weights for the Haar wavelet transform, i. e. $\mathbf{b }_H[t] > \lambda$ iff Haar wavelet regression with threshold $\lambda$ contains a discontinuity between $t-1$ an *t*, and therefore a block boundary in Haar wavelet compression. This is the case if the absolute value of any coefficient of wavelets who have any of their discontinuities at *t* as above the threshold, so we define, for any $t = b^\pm _{j,k} \in [0, T)$,

1 $$\begin{aligned} \mathbf{b }_H[t]:=\max _{j,k} \left\{ \left|{} \left\langle {\varvec{\psi }} _{j,k}, \mathbf{y } \right\rangle \right| \,\big |\, t\in \left\{ b^+_{j,k}, b^\pm _{j,k}, b^-_{j,k} \right\} \right\} \end{aligned}$$

for $t>0$ or $b^-_{j,k} < T$. Additionally, there is always a block boundary before the first position, so $\mathbf{b }_H[0]:=\infty$. Furthermore, if *T* is not a power of 2, some wavelets have incomplete support. As their magnitude is unknown without padding the data, we assume that their detail coefficient is potentially larger than any threshold, inducing a breakpoint at the central discontinuity, so $\mathbf{b }_H\left[ b^\pm _{j,k}\right] :=\infty$ for $b^-_{j,k}\ge T$. A breakpoint array initialized with these weights is called a *Haar breakpoint array*.

We will show that $\mathbf{b }_H$ can be computed in-place and in linear time. For that purpose, we first define the *maxlet array* as a generalization of the Haar transform to arbitrary data sizes and absolute values: For $b^\pm _{j,k} \in [0, T)$, let

$$\mathbf{b }_M\left[ b^\pm _{j,k}\right] = {\left\{ \begin{array}{ll} \infty & \quad t=0 \vee b^-_{j,k}\ge T \\ \left| \left\langle {\varvec{\psi }} _{j,k}, \mathbf{y } \right\rangle \right| & \quad t>0 \vee b^-_{j,k} < T. \end{array}\right. }$$

We later define the *Haar boundary transform* to compute $\mathbf{b }_H$ from $\mathbf{b }_M$. In order to compute $\mathbf{b }_M$ in-place, we cannot use the pyramid algorithm as in [6], since it requires padding of the data to a size $T'\in 2^{\mathbb {N}}$, $T\le T'\le 2T$, as well as an auxiliary array of size $T'$, thereby increasing the memory by up to a factor of 4. Instead, we use a more recent in-place calculation of the Haar wavelet transform based on the lifting scheme [13, 22]. It is based on the following recursions:

$$\begin{aligned}& c_{j,k} :={\left\{ \begin{array}{ll} \mathbf{y }[k] & \quad j=0\\ \sum _{t=b^+_{j,k}}^{b^-_{j,k} -1} \mathbf{y }[t] = c_{j-1, 2k} + c_{j-1, 2k+1} & \quad j>0\text {, and}\\ \end{array}\right. }\\& d_{j,k} :=\frac{1}{\sqrt{2^j}}\left( c_{j-1, 2k} + c_{j-1, 2k+1}\right) . \end{aligned}$$

**Fig. 5:**
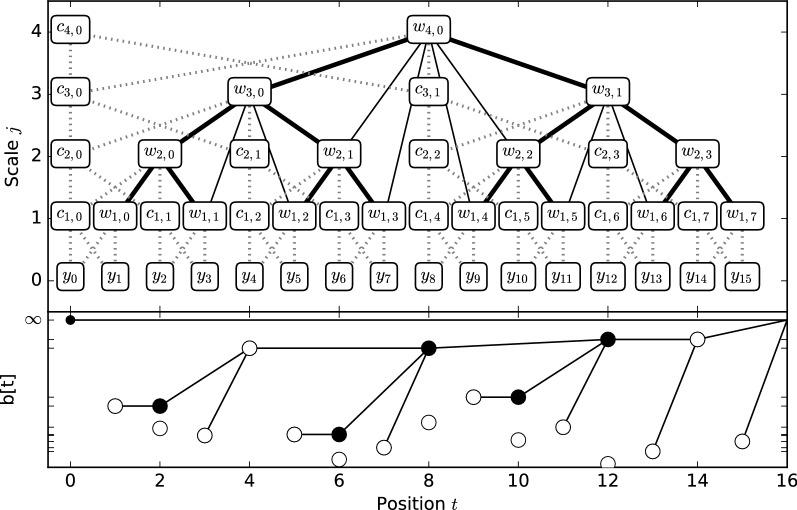
Illustration of the various algorithms necessary to create the Haar breakpoint array in-place. The top figure represents the transformation of an input array $\mathbf{y }$ at level 0 into various other forms. The terms $c_{j,k}$ and $w_{j,k}$ represent values associated with the scale and detail coefficients of the wavelet transform, respectively. The wavelet tree (bold lines) represents the nested nature of the support intervals: the horizontal position of $\psi _{j,k}$ represents the position *t* of central discontinuity $\mathbf{b }_{j,k}^\pm$ of ${\varvec{\psi }} _{j,k}$, and its vertical position represents the resolution level *i*. The support interval for each wavelet corresponds to all descendants at level 0. The tree nodes contain the output arrays of the various transforms. Dotted lines indicate the recursive relations in the lifting scheme, as used by the in-place Haar wavelet transform and the maxlet transform. The solid lines (including tree edges) indicate the dependencies in the Haar boundary transform. In the bottom figure, white bullets represent maxlet coefficients, black bullets represent their changed values after the Haar boundary transform, and lines indicate breakpoint array pointers

These relations are illustrated in Fig. 5 using dotted edges, with $d_{j,k}=w_{j,k}$ and $c_{0,k} = y_{k} = \mathbf{y }[k]$. By storing $c_{j,k}$ at index $b^+_{j,k}$ and $d_{j,k}$ at index $b^\pm _{j,k}$, this yields a simple in-place algorithm which never overwrites $d_{j,k}$ once it is calculated. Notice that detail coefficients $d_{j,k}$ are stored at the position $b^\pm _{j,k}$ corresponding to the central discontinuity in their corresponding wavelet, and that this corresponds to an in-order DFS layout of the wavelet tree without the leaves corresponding to the input data, with the leftmost leaf at index 1 (Fig. 5, bold lines); the tree is created from the leaves up, and from left to right. A straightforward modification of the lifting scheme to calculate $\mathbf{b }_M$ is shown in Algorithm 2, where line 13 is changed to yield the absolute value, and lines 9, 14 and 15 are added to ensure $\mathbf{b }_H\left[ b^\pm _{j,k}\right] :=\infty$ for $b^-_{j,k}\ge T$.

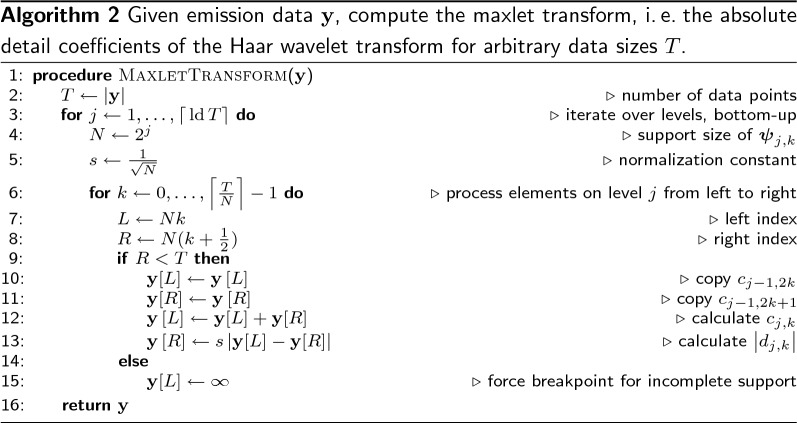



To derive Haar breakpoint weight from the maxlet transform, we introduce the *Haar boundary transform* (Algorithm 3), which performs the necessary maximum computations for Eq. 1 in-place and in linear time *O*(*T*). In Fig. 5 (top), the set of nodes considered in Eq. 1 are the direct descendants of a node along the solid lines. Algorithm 3 is simple: it iterates over the scales *j* in a top-down fashion (Fig. 5), and writes the maxima of all required nodes at lower levels $\ell \le j$ to the current array position. Since it never reads values from levels $>j$, no extra memory is required, and the algorithm is in-place. Since any node is considered at most twice for updating a node on a higher level, the running time of the Haar boundary transform is also linear, *O*(*T*).

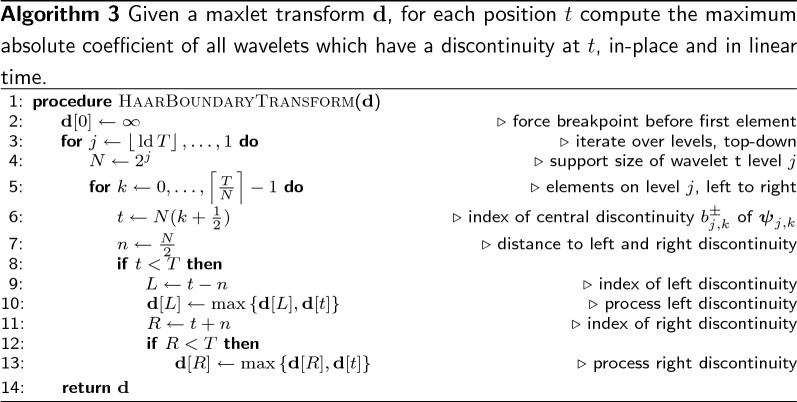



### Compressed marginal records

In order to keep track of the states sampled for each position during Gibbs sampling, we require the following data structure:

#### **Definition 2.3**

(*Marginal records*) Let $t\in [0, \ldots , T)$, $s_{\max }$ the largest state sampled during FBG, and $s\in [0, \ldots , s_{\max }]$. A *marginal record* is a data structure which allows to store and query the number of times state *s* was observed at data index *t*.

The previous solution to recording marginal state counts was inefficient. Since nodes in the wavelet tree corresponded to compression blocks, counts were stored directly in the nodes. For *n* latent HMM states, this required allocation of 2*Tn* array elements, which was wasteful since the quick convergence of HaMMLET meant that many blocks would never be sampled, or only be assigned to a small subset of CNV states. Such a preallocation approach also requires the number of states to be known in advance, and precludes further extensions to priors on the state number such as the Dirichlet Process. Though we resorted to dynamic allocation, the necessary variables for housekeeping still incurred large overhead.

**Fig. 6:**
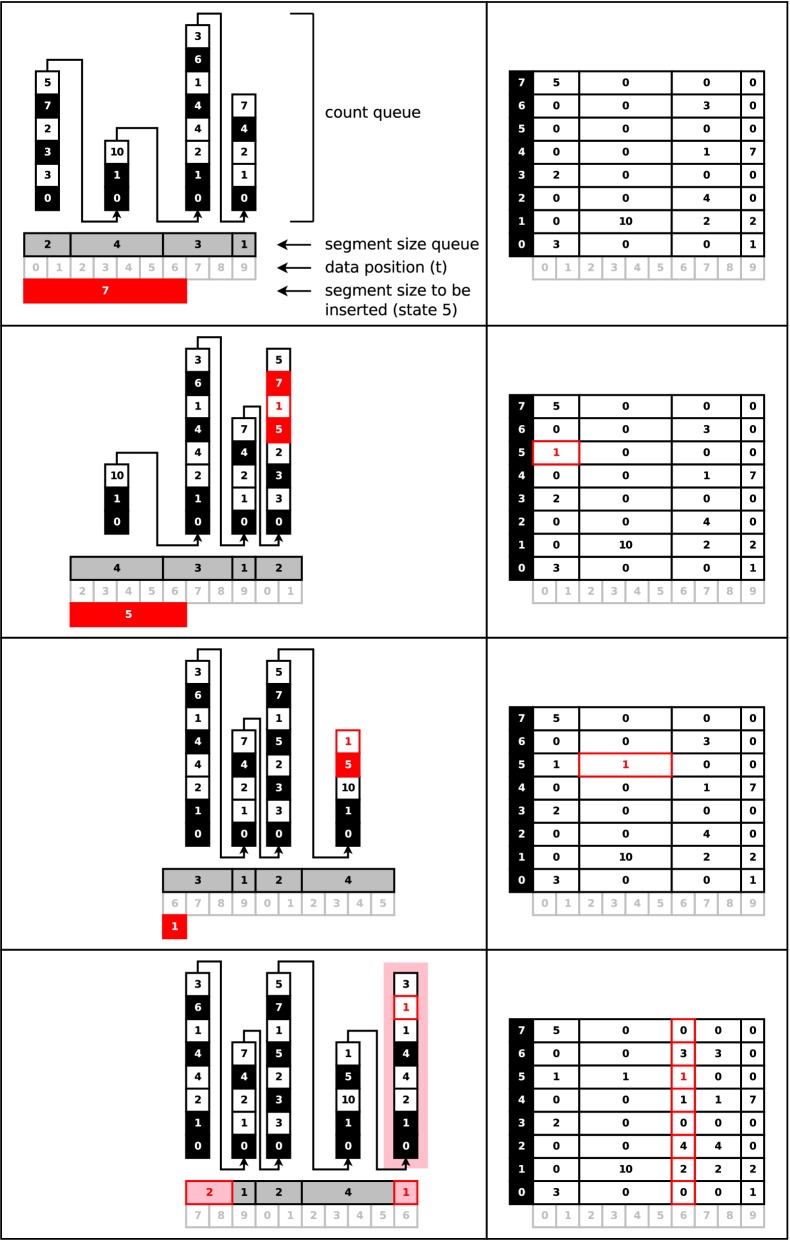
A small three-step example of recording marginal counts using Algorithm 4. Starting at position $t = 0$, 7 observations of state 5 are inserted. In the count queue, black boxes indicate that state counts of zero have been skipped; those numbers encode the next higher state that has a non-zero count. White boxes indicate the counts for the state. For instance, the right-most part of the count queue in the top subfigure is stored as $(0, -1, -2, 4, -7)$, indicating that there is 1 count for state 0, 2 counts for state 1, and 7 counts for state 4. The segment starts at position $t=9$, and has a length of 1. Note that 0 is used to mark the start of a new segment. Each segment has a total of 10 counts already recorded. Arrows indicate contiguous elements in the count queue. With every iteration, a segment is moved to the back with the new state count included. Note that in the last iteration, the segment $t=6, \dots , 8$ is split. After finishing this step, the next count would be recorded starting at position $t=7$. Notice how each run of zeros in the state queue is represented by a single number, thus allowing for arbitrarily large state indices without much overhead

For static compression blocks, marginals can simply be stored in a set of arrays with an additional array containing block sizes, essentially a run-length encoding (RLE), as illustrated by the right column of Fig. 6. This approach however is complicated by the use of dynamic compression: at each new iteration, a different block structure is created, which requires existing RLE segments to be split into multiple parts, each of which will have counts for a different state added. This could be solved trivially using a linked list implementation, in which new segments are inserted with the appropriate updates of its neighbors size. This approach is obviously wasteful.

To get around these issues, we developed an encoding for marginal records that stores counts sequentially in a vector of integers in a highly compressed fashion with minimum overhead. Adding records for run-length encoded state sequences is performed using a queue with iterator access to its front elements, such as implemented by the C++ STL deque, and requires a single pass over the state records and is therefore linear. The memory overhead is 2 bytes per segment, plus one bit for every 32 integers. Encoding for marginal counts for a single position is performed using a sequence $\mathbf{c }$ of signed integers. A negative number is used to store the counts for a state. The state *s*(*i*) of a position *i* is recursively defined as

$$\begin{aligned} s(0) = 0\quad \quad s(i) :={\left\{ \begin{array}{ll} s(i-1) &{} c[i-1]<0\\ \mathbf{c }[i-1] &{} c[i-1]>0. \end{array}\right. } \end{aligned}$$

Positive entries are called *index values*. We further require that all index values must be in strictly increasing order, and that no unnecessary index is used, i. e. we require $\forall \mathbf{c }[i]>0: s(i-1)+1 < \mathbf{c }[i]$. In other words, runs of states having observed counts are represented as runs of negative numbers, and runs of zero-counts are represented as a single number indicating the state label of the next higher state with non-zero counts. For instance, the count vector (2, 0, 0, 8, 1, 4, 0, 0, 0, 0, 5, 0, 0, 0, 0, 0, 0, 0, 0) would be encoded as $(-2, 3, -8, -1, -4, 9, -5)$, and the corresponding states are (0, 1, 3, 4, 5, 6, 9), though 1 and 6 are somewhat inconsequential as they have no counts associated with them; note that the decision to use negative signs for counts instead of index values is arbitrary in principle, but leads to using fewer negations in the implementation. In settings where quick convergence is expected, the number of zeros is expected to be high, leading to good compression under this scheme. In general, assume that the marginals contain *M* distinct segments after running FBG, and the HMM has *S* states. Then, the queue can contain no more than $(2S + 1)M$ entries: for each segment, one zero to mark the beginning of a segment, and up to one positive and negative value per state. If the number of latent HMM states is limited to *S*, then there can be no more than *S* non-zero entries per segment. Hence, for reasonably high compression ratios, this amounts to small memory usage. For instance, at a compression ratio of 300 for a human genome at base-level resolution and 10 latent HMM states, marginal records using 2-byte signed integers require less than 234  MB. In practice, not every segment will contain 11 values, due to fast convergence, and the numbers get even smaller. Compared to the storage requirements of the block generator, this is negligible.

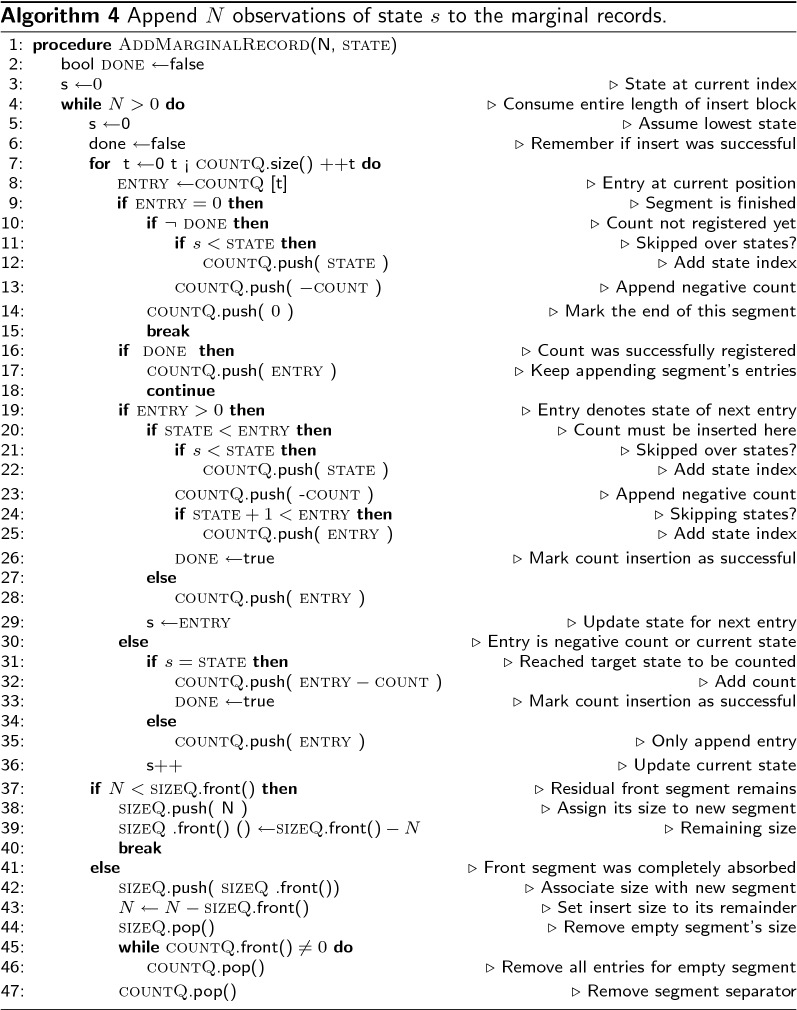



## Results and discussion

In order to verify that the higher compression did not adversely affect the segmentation quality, we re-ran the evaluation on the 129,000 simulated datasets in [6] using our new implementation of HaMMLET. The F-measures and plots are virtually identical to Fig.  5 in that paper, and are therefore not shown here (see Web Supplement).

In the following subsections, we present a case study of CNV inference on differential WGS read depth data using HaMMLET with the Haar breakpoint array.

### Experiment background

The domestication of a handful of animal species, starting in the early holocene, has played a crucial role in the development of complex human societies [23]. While we have learned a great deal about when and where animal domestication occurred, the genetic changes that underlie the phenotypic differences between domestic animals and their wild progenitors remain relatively unknown. It has been observed that domestic animal species tend to share a suite of behavioral, physiological and morphological traits that are absent or rarely observed in their wild progenitors [24, 25]. These traits include changes in pigmentation, craniofacial anatomy, hormonal levels, seasonal reproduction cycles and increased docility [26]. This suite of changes is referred to as the “domestication syndrome”. A long-standing question in evolutionary biology is whether these convergent changes are the result of genetic drift, artificial selection by humans for each individual trait, or pleiotropic effects of selection for a few or even a single trait. A proponent of the latter hypothesis was the Academician Dmitry K. Belyaev. He hypothesised that selection for tameness at the start of the domestication process had pleiotropic effects that explained many of the features of the domestication syndrome. To test his hypothesis, he began a program of experimental domestication of the silver fox (*Vulpes vulpes*) in Novosibirsk, Siberia in 1959. Foxes obtained for fur farms were selectively bred for their behavioral response to an approaching human. One line of foxes was bred for tame behavior towards humans while a control line was selected for a fearful and aggressive response towards humans, to maintain the wild-type behavior despite being maintained in captive conditions. After just a few generations of selective breeding the tame line began to show many of the traits associated with the domestication syndrome, including changes in pigmentation, morphology and behavior [27–29].

The same experimental setup of artificially selecting two lines, one for tame and one for fearful and aggressive behavior towards humans was also repeated by the same research group in the brown Norway rat (*Rattus norvegicus*) with similar results [30]. These results seem to confirm Belyaev’s hypothesis that selection for tameness alone could explain many of the features of the domestication syndrome. However, the specific genetic changes that underlie these changes remain unknown. Knowledge of the genetic variants that have been selected in these lines could lead to mechanistic insights into the domestication process. Genomic structural variants are of particular interest as they are known to have played a role in the adaptation of other domestic animals [31] and structural variants that affect multiple functional genomic loci are one possible explanation for the rapid response to selection observed in these lines. To address this issue we analysed whole-genome data that was generated from multiple individuals from the tame and aggressive lines of rats.

### Sample origins and data generation

DNA samples were obtained from two rat lines originating from a shared wild source population and subsequently maintained in isolation and divergently selected for $\sim$70 generations for their behavioral response to humans. 20 samples were obtained from the tame line, which has been selected for a reduced fear response towards an approaching human hand. 20 samples were obtained from the aggressive line, which has been selected for an increase in fearful and aggressive behavior towards an approaching human hand. DNA extraction was carried out at the Institute of Cytology and Genetics, the Siberian Branch of the Russian Academy of Sciences, Novosibirsk and at the Max Planck Institute for Evolutionary Anthropology (MPI-EVA), Germany.

For all samples, sequencing libraries were generated consisting of 125 bp double-indexed paired-end reads. Samples were pooled into a single library in order to avoid any batch effects during sequencing. Sequencing was performed on a combination of the Illumina Genome Analyzer II and High-Seq platforms. Library preparation and sequencing was carried out at the MPI-EVA. The rats have a mean coverage of $\sim$4× per individual. Base calling was done using freeIbis [32]. Adapters were removed and potentially chimeric sequences flagged using leeHom with default parameters [33]. Reads were demultiplexed using deML using default quality thresholds [34]. Reads were then mapped to the *Rattus norvegicus* reference assembly rno5, using the BWA with default parameters [35]. Duplicate read removal was performed with Picard (http://broadinstitute.github.io/picard/). Local indel realignment was performed using GATK [36]. Lowest mapping positions were recorded for each read, and their counts were accumulated. Start counts for the tame population were subtracted from their counterparts in the aggressive population, yielding 1,880,703,547 data points. Due to the low coverage, the data showed highly discrete noise, and hence the data was averaged over non-overlapping windows of 20 positions to approximate Gaussian noise, resulting in 94,035,178 input positions. We then ran HaMMLET with 8 CNV states and automatic priors, see [6].

### Computational benchmarks

On a computer with Intel Xeon CPU E7-8890 v4 (2.20 GHz) and 1 TB RAM, running Ubuntu 14.04.5 LTS, full Bayesian inference with HaMMLET for 200 iterations with a burn-in of 1800 for an 8-state-model required 3 min 41 s and 1.3 GB RAM on a single core. By comparison, the previously published version of HaMMLET took 1 h 5 min 27 s, using 40 GB RAM, a 17.8-fold speedup.

**Fig. 7:**
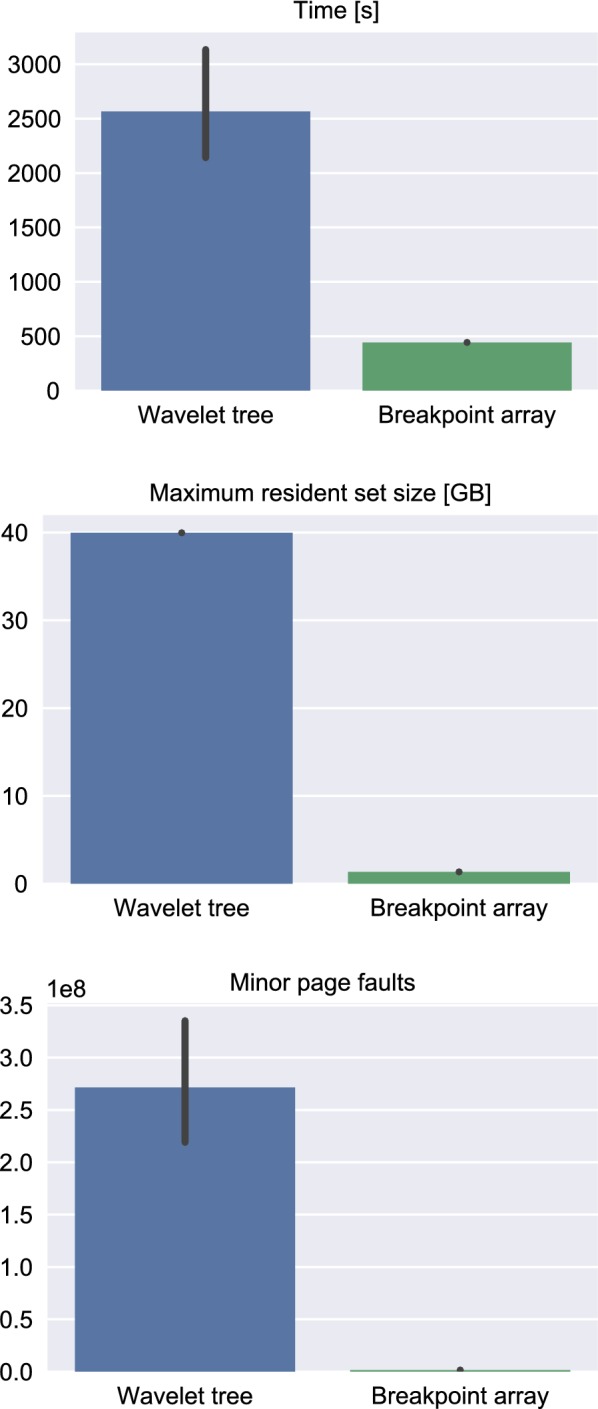
Comparison of benchmarks for running time, memory usage and cache behavior between the old and new versions of HaMMLET on the rat population WGS data set. The new approach yields a 17.8-fold speedup and 32.2-fold memory reduction. Notice that the number of minor page faults decreases by two orders of magnitude, indicating much better cache behavior due to the use of new data structures and an improved implementation. The number of major page faults is zero in both implementations. The wavelet tree benchmarks also contain one outlier with 2.4 billion page faults and 6.4 h runtime, likely due to undercompression. No such anomaly was observed for the breakpoint array

For a broader evaluation, we have created 100 replicates of the data by splitting it into 2500 chunks of equal sizes, which we then permuted randomly. We measured the memory usage (maximum resident set size), running time as well as cache behavior (minor page faults), see the boxplots in Fig. 7). The smaller savings in runtime compared to the original data can be attributed to the fact that permutation of the data is likely to disrupt long highly compressible sections of the data.

While the RAM usage remains almost constant among replicates within each implementation, we noticed that runtime and cache behavior varied widely in the old, but not the new implementation. We attribute this to the fact that the old compression scheme is suboptimal, yielding smaller blocks and hence more randomized assignment to states, leading to slower mixing properties of the Gibbs sampler. Notice that the data contains outliers which are likely to result from sampling small emission variances due to short compression blocks.

### Biological results

We consider all genomic segments with an absolute state mean $\ge 1$ as containing putative structural variation segregating between the tame and aggressive rat lines. This results in 10,083,374 regions with a mean size of 407 base pairs. We identify all genes that are within or overlap these regions by $\ge 1$ base pair using Ensembl’s Variant Effect Predictor [37]. We find 1036 genes with at least partial overlap with these regions.

To investigate the potential phenotypic consequences of these structural variants we performed GO gene enrichment analysis using the software Webgestalt [38, 39]. We tested for enrichment of GO categories using all genes overlapping these structural variants using all genes in the rat genome as background. We consider as significantly enriched all pathways with p-value $<0.05$ after using the Benjamini and Hochberg procedure to correct for multiple hypothesis testing [40]. We identify many significantly enriched pathways (Additional file 1: Table S1). We now briefly discuss some of these pathways and the genes within them and how they may inform us about the genetic changes underlying the phenotypic differences between these lines.

The most significantly enriched pathway is “Synapse assembly” (p-value = 0.0028), with five genes that are in putative structural variants segregating between the tame and aggressive rat lines. Some of these genes are associated with phenotypes that may be involved in the behavioral differences observed between the tame and aggressive rat lines. For example, one of the genes is the neuronal cadherin gene Cdh2. Missense mutations in this gene are associated with obsessive-compulsive behavior and Tourette disorder phenotypes in humans [41] and this gene has been associated with anxiety in mice [42]. Another gene encodes the ephrin receptor Ephb1. The ephrin receptor-ligand system is involved in the regulation of several developmental processes in the nervous system. Notably, mice with null mutations for this gene exhibit neuronal loss in the substantia nigra and display spontaneous locomotor hyperactivity [43]. This is interesting given that the tame and aggressive rats have differences in their activity in an open-field test [30].

We also observe multiple additional enriched pathways involved in neuronal development and function, e.g. “transmission of nerve impulse”, “regulation of neurological system process”, “dendrite morphogenesis”. Therefore, we suspect that many of these segregating structural variants may have been targeted by selection and are contributing the phenotypic differences between these lines. Future study of the variants identified here may lead to insights into the domestication process. A more detailed evaluation of our finding will be published elsewhere. Plots of segmentation results for the entire genome can be found in the web supplement at https://schlieplab.org/Supplements/rats/.

## Conclusion

We have presented an new wavelet compression scheme for HaMMLET. The compression is optimal in that it does not introduce unnecessary block boundaries in addition to the wavelet regression discontinuities. This leads to much more stable benchmarks and reliable performance. Additional improvements, such as a memory-efficient data structure for marginal state records, allow for Bayesian inference of a hidden Markov model of genome-sized data, such as for CNV calling, on standard consumer hardware. Future applications include inference on multivariate data. By computing detail coefficients in post-order DFS across all dimensions simultaneously, and the maxlet transform has a straightforward generalization to higher dimensions with only $O(\log T)$ overhead, instead of the naive $\Theta (T)$ incurred by aggregating maxima in a second array.

## Availability and requirements

**Project name:** HaMMLET
**Project home page:** https://schlieplab.org/Software/HaMMLET/
**Operating system:** Platform-independent
**Programming language:** C++
**Other requirements:** C++11-compliant compiler. For plotting: Python 2.7, Matplotlib
**License:** GNU GPL.

## Additional file

**Additional file 1: Table S1.** Significantly enriched GO categories.

## Data Availability

https://schlieplab.org/Supplements/rats/.
