# Supplementary material for: Bayesian localization of CNV candidates in WGS data within minutes
Source: Algorithms Mol Biol. 2019 Sep 23;14:20. doi: 10.1186/s13015-019-0154-7 (PMC6757390; doi:10.1186/s13015-019-0154-7)
Supplement: Supplementary file 1 — Additional file 1: Table S1. Significantly enriched GO categories. [file 13015_2019_154_MOESM1_ESM.pdf]

Table S1. Significantly enriched GO categories.

The results for each enriched GO category are listed in this table. For each GO category, the first row lists its sub-root (biological process, molecular function, or cellular component), category name, and corresponding GO ID. The second row lists number of reference genes in the category (C), number of genes in the gene set and also in the category (O), expected number in the category (E), Ratio of enrichment (R), p value from hypergeometric test (rawP), and p value adjusted by the multiple test adjustment (adjP). Finally, genes in the category are listed. For each gene, the table lists the user uploaded ID and value (optional), Entrez ID, Ensembl Gene Stable ID, Gene symbol, and description.

biological process	synapse assembly	GO:0007416			
C=65;O=5;E=0.24;R=20.88;rawP=3.88e-06;adjP=0.0028					
Cacna1a	NA	Cacna1a	calcium channel, voltage-dependent, P/Q type, alpha 1A subunit	25398	ENSRNOG00000002559
Cdh2	NA	Cdh2	cadherin 2	83501	ENSRNOG00000015602
Ephb1	NA	Ephb1	Eph receptor B1	24338	ENSRNOG00000007865
Nrg1	NA	Nrg1	neuregulin 1	112400	ENSRNOG00000010392
Ptk2	NA	Ptk2	PTK2 protein tyrosine kinase 2	25614	ENSRNOG00000007916

biological process	regulation of cell communication	GO:0010646			
C=1792;O=17;E=6.60;R=2.57;rawP=0.0001;adjP=0.0064					
Cacna1a	NA	Cacna1a	calcium channel, voltage-dependent, P/Q type, alpha 1A subunit	25398	ENSRNOG00000002559
Cdh2	NA	Cdh2	cadherin 2	83501	ENSRNOG00000015602
Ephb1	NA	Ephb1	Eph receptor B1	24338	ENSRNOG00000007865
Myo9b	NA	Myo9b	myosin IXb	25486	ENSRNOG00000016256
Gria4	NA	Gria4	glutamate receptor, ionotropic, AMPA 4	29629	ENSRNOG00000006957
Camk2g	NA	Camk2g	calcium/calmodulin-dependent protein kinase II gamma	171140	ENSRNOG00000009783
Plau	NA	Plau	plasminogen activator, urokinase	25619	ENSRNOG00000010516
Sall1	NA	Sall1	sal-like 1 (Drosophila)	307740	NULL
Cyth1	NA	Cyth1	cytohesin 1	116691	ENSRNOG00000043381
Epha4	NA	Epha4	Eph receptor A4	316539	ENSRNOG00000013213
Ppp3cb	NA	Ppp3cb	protein phosphatase 3, catalytic subunit, beta isozyme	24675	ENSRNOG00000007757
Scoc	NA	Scoc	short coiled-coil protein	364981	ENSRNOG00000003853
Egfr	NA	Egfr	epidermal growth factor receptor	24329	ENSRNOG00000004332
Nrg1	NA	Nrg1	neuregulin 1	112400	ENSRNOG00000010392
Ptk2	NA	Ptk2	PTK2 protein tyrosine kinase 2	25614	ENSRNOG00000007916
Rheb	NA	Rheb	Ras homolog enriched in brain	26954	NULL
Uaca	NA	Uaca	uveal autoantigen with coiled-coil domains and ankyrin repeats	315732	NULL

biological process	localization of cell	GO:0051674			
C=807;O=11;E=2.97;R=3.70;rawP=0.0001;adjP=0.0064					
Cdh2	NA	Cdh2	cadherin 2	83501	ENSRNOG00000015602
Ddr2	NA	Ddr2	discoidin domain receptor tyrosine kinase 2	685781	ENSRNOG00000002881
Ephb1	NA	Ephb1	Eph receptor B1	24338	ENSRNOG00000007865
Epha4	NA	Epha4	Eph receptor A4	316539	ENSRNOG00000013213
Myo9b	NA	Myo9b	myosin IXb	25486	ENSRNOG00000016256
Egfr	NA	Egfr	epidermal growth factor receptor	24329	ENSRNOG00000004332
Ptk2	NA	Ptk2	PTK2 protein tyrosine kinase 2	25614	ENSRNOG00000007916
Nrg1	NA	Nrg1	neuregulin 1	112400	ENSRNOG00000010392
Plau	NA	Plau	plasminogen activator, urokinase	25619	ENSRNOG00000010516
Dcdc2	NA	Dcdc2	doublecortin domain containing 2	291130	ENSRNOG00000017511
Vcl	NA	Vcl	vinculin	305679	ENSRNOG00000010765

biological process	establishment of cell polarity	GO:0030010			
C=65;O=4;E=0.24;R=16.70;rawP=9.37e-05;adjP=0.0064					
Sdccag8	NA	Sdccag8	serologically defined colon cancer antigen 8	305002	ENSRNOG00000004181
Ephb1	NA	Ephb1	Eph receptor B1	24338	ENSRNOG00000007865
Cyth1	NA	Cyth1	cytohesin 1	116691	ENSRNOG00000043381
Myo9b	NA	Myo9b	myosin IXb	25486	ENSRNOG00000016256

biological process	synapse organization	GO:0050808			
C=128;O=5;E=0.47;R=10.60;rawP=0.0001;adjP=0.0064					
Cacna1a	NA	Cacna1a	calcium channel, voltage-dependent, P/Q type, alpha 1A subunit	25398	ENSRNOG00000002559
Cdh2	NA	Cdh2	cadherin 2	83501	ENSRNOG00000015602
Ephb1	NA	Ephb1	Eph receptor B1	24338	ENSRNOG00000007865
Nrg1	NA	Nrg1	neuregulin 1	112400	ENSRNOG00000010392
Ptk2	NA	Ptk2	PTK2 protein tyrosine kinase 2	25614	ENSRNOG00000007916

biological process	regulation of transmission of nerve impulse	GO:0051969			
C=259;O=7;E=0.95;R=7.34;rawP=4.24e-05;adjP=0.0064					
Cacna1a	NA	Cacna1a	calcium channel, voltage-dependent, P/Q type, alpha 1A subunit	25398	ENSRNOG00000002559
Egfr	NA	Egfr	epidermal growth factor receptor	24329	ENSRNOG00000004332
Cdh2	NA	Cdh2	cadherin 2	83501	ENSRNOG00000015602
Gria4	NA	Gria4	glutamate receptor, ionotropic, AMPA 4	29629	ENSRNOG00000006957

Camk2g	NA	Camk2g	calcium/calmodulin-dependent protein kinase II gamma	171140	ENSRNOG00000009783
Ptk2	NA	Ptk2	PTK2 protein tyrosine kinase 2	25614	ENSRNOG00000007916
Rheb	NA	Rheb	Ras homolog enriched in brain	26954	NULL

biological process	cell migration	GO:0016477			
C=750;O=11;E=2.76;R=3.98;rawP=7.13e-05;adjP=0.0064					
Cdh2	NA	Cdh2	cadherin 2	83501	ENSRNOG00000015602
Ddr2	NA	Ddr2	discoidin domain receptor tyrosine kinase 2	685781	ENSRNOG00000002881
Ephb1	NA	Ephb1	Eph receptor B1	24338	ENSRNOG00000007865
Epha4	NA	Epha4	Eph receptor A4	316539	ENSRNOG00000013213
Myo9b	NA	Myo9b	myosin IXb	25486	ENSRNOG00000016256
Egfr	NA	Egfr	epidermal growth factor receptor	24329	ENSRNOG00000004332
Ptk2	NA	Ptk2	PTK2 protein tyrosine kinase 2	25614	ENSRNOG00000007916
Nrg1	NA	Nrg1	neuregulin 1	112400	ENSRNOG00000010392
Plau	NA	Plau	plasminogen activator, urokinase	25619	ENSRNOG00000010516
Dcdc2	NA	Dcdc2	doublecortin domain containing 2	291130	ENSRNOG00000017511
Vcl	NA	Vcl	vinculin	305679	ENSRNOG00000010765

biological process	regulation of neurological system process	GO:0031644			
C=283;O=7;E=1.04;R=6.71;rawP=7.43e-05;adjP=0.0064					
Cacna1a	NA	Cacna1a	calcium channel, voltage-dependent, P/Q type, alpha 1A subunit	25398	ENSRNOG00000002559
Egfr	NA	Egfr	epidermal growth factor receptor	24329	ENSRNOG00000004332
Cdh2	NA	Cdh2	cadherin 2	83501	ENSRNOG00000015602
Gria4	NA	Gria4	glutamate receptor, ionotropic, AMPA 4	29629	ENSRNOG00000006957
Camk2g	NA	Camk2g	calcium/calmodulin-dependent protein kinase II gamma	171140	ENSRNOG00000009783
Ptk2	NA	Ptk2	PTK2 protein tyrosine kinase 2	25614	ENSRNOG00000007916
Rheb	NA	Rheb	Ras homolog enriched in brain	26954	NULL

biological process	protein autophosphorylation	GO:0046777			
C=182;O=6;E=0.67;R=8.95;rawP=5.24e-05;adjP=0.0064					
Egfr	NA	Egfr	epidermal growth factor receptor	24329	ENSRNOG00000004332
Ddr2	NA	Ddr2	discoidin domain receptor tyrosine kinase 2	685781	ENSRNOG00000002881
Ephb1	NA	Ephb1	Eph receptor B1	24338	ENSRNOG00000007865
Epha4	NA	Epha4	Eph receptor A4	316539	ENSRNOG00000013213
Camk2g	NA	Camk2g	calcium/calmodulin-dependent protein kinase II gamma	171140	ENSRNOG00000009783
Ptk2	NA	Ptk2	PTK2 protein tyrosine kinase 2	25614	ENSRNOG00000007916

biological process	cell motility	GO:0048870			
C=807;O=11;E=2.97;R=3.70;rawP=0.0001;adjP=0.0064					
Cdh2	NA	Cdh2	cadherin 2	83501	ENSRNOG00000015602
Ddr2	NA	Ddr2	discoidin domain receptor tyrosine kinase 2	685781	ENSRNOG00000002881
Ephb1	NA	Ephb1	Eph receptor B1	24338	ENSRNOG00000007865
Epha4	NA	Epha4	Eph receptor A4	316539	ENSRNOG00000013213
Myo9b	NA	Myo9b	myosin IXb	25486	ENSRNOG00000016256
Egfr	NA	Egfr	epidermal growth factor receptor	24329	ENSRNOG00000004332
Ptk2	NA	Ptk2	PTK2 protein tyrosine kinase 2	25614	ENSRNOG00000007916
Nrg1	NA	Nrg1	neuregulin 1	112400	ENSRNOG00000010392
Plau	NA	Plau	plasminogen activator, urokinase	25619	ENSRNOG00000010516
Dcdc2	NA	Dcdc2	doublecortin domain containing 2	291130	ENSRNOG00000017511
Vcl	NA	Vcl	vinculin	305679	ENSRNOG00000010765
biological process	central nervous system neuron axonogenesis	GO:0021955			
C=28;O=3;E=0.10;R=29.08;rawP=0.0001;adjP=0.0064					
Ephb1	NA	Ephb1	Eph receptor B1	24338	ENSRNOG00000007865
Epha4	NA	Epha4	Eph receptor A4	316539	ENSRNOG00000013213
Ptk2	NA	Ptk2	PTK2 protein tyrosine kinase 2	25614	ENSRNOG00000007916
biological process	regulation of synaptic transmission	GO:0050804			
C=233;O=6;E=0.86;R=6.99;rawP=0.0002;adjP=0.0068					
Cacna1a	NA	Cacna1a	calcium channel, voltage-dependent, P/Q type, alpha 1A subunit	25398	ENSRNOG00000002559
Egfr	NA	Egfr	epidermal growth factor receptor	24329	ENSRNOG00000004332
Gria4	NA	Gria4	glutamate receptor, ionotropic, AMPA 4	29629	ENSRNOG00000006957
Camk2g	NA	Camk2g	calcium/calmodulin-dependent protein kinase II gamma	171140	ENSRNOG00000009783
Ptk2	NA	Ptk2	PTK2 protein tyrosine kinase 2	25614	ENSRNOG00000007916
Rheb	NA	Rheb	Ras homolog enriched in brain	26954	NULL
biological process	dendrite morphogenesis	GO:0048813			
C=76;O=4;E=0.28;R=14.29;rawP=0.0002;adjP=0.0068					
Cacna1a	NA	Cacna1a	calcium channel, voltage-dependent, P/Q type, alpha 1A subunit	25398	ENSRNOG00000002559
Ephb1	NA	Ephb1	Eph receptor B1	24338	ENSRNOG00000007865
Epha4	NA	Epha4	Eph receptor A4	316539	ENSRNOG00000013213

Dcdc2	NA	Dcdc2	doublecortin domain containing 2	291130	ENSRNOG00000017511
biological process	transmission of nerve impulse	GO:0019226			
C=575;O=9;E=2.12;R=4.25;rawP=0.0002;adjP=0.0068					
Cacna1a	NA	Cacna1a	calcium channel, voltage-dependent, P/Q type, alpha 1A subunit	25398	ENSRNOG00000002559
Cdh2	NA	Cdh2	cadherin 2	83501	ENSRNOG00000015602
Ephb1	NA	Ephb1	Eph receptor B1	24338	ENSRNOG00000007865
Egfr	NA	Egfr	epidermal growth factor receptor	24329	ENSRNOG00000004332
Gria4	NA	Gria4	glutamate receptor, ionotropic, AMPA 4	29629	ENSRNOG00000006957
Ptk2	NA	Ptk2	PTK2 protein tyrosine kinase 2	25614	ENSRNOG00000007916
Nrg1	NA	Nrg1	neuregulin 1	112400	ENSRNOG00000010392
Camk2g	NA	Camk2g	calcium/calmodulin-dependent protein kinase II gamma	171140	ENSRNOG00000009783
Rheb	NA	Rheb	Ras homolog enriched in brain	26954	NULL
biological process	monovalent inorganic anion homeostasis	GO:0055083			
C=6;O=2;E=0.02;R=90.48;rawP=0.0002;adjP=0.0068					
Cacna1a	NA	Cacna1a	calcium channel, voltage-dependent, P/Q type, alpha 1A subunit	25398	ENSRNOG00000002559
Ptk2	NA	Ptk2	PTK2 protein tyrosine kinase 2	25614	ENSRNOG00000007916
biological process	chloride ion homeostasis	GO:0055064			
C=6;O=2;E=0.02;R=90.48;rawP=0.0002;adjP=0.0068					
Cacna1a	NA	Cacna1a	calcium channel, voltage-dependent, P/Q type, alpha 1A subunit	25398	ENSRNOG00000002559
Ptk2	NA	Ptk2	PTK2 protein tyrosine kinase 2	25614	ENSRNOG00000007916
biological process	cellular chloride ion homeostasis	GO:0030644			
C=6;O=2;E=0.02;R=90.48;rawP=0.0002;adjP=0.0068					
Cacna1a	NA	Cacna1a	calcium channel, voltage-dependent, P/Q type, alpha 1A subunit	25398	ENSRNOG00000002559
Ptk2	NA	Ptk2	PTK2 protein tyrosine kinase 2	25614	ENSRNOG00000007916
biological process	biological adhesion	GO:0022610			
C=695;O=10;E=2.56;R=3.91;rawP=0.0002;adjP=0.0068					
Stab2	NA	Stab2	stabilin 2	282580	NULL
Cdh2	NA	Cdh2	cadherin 2	83501	ENSRNOG00000015602

Ephb1	NA	Ephb1	Eph receptor B1	24338	ENSRNOG00000007865
Cyth1	NA	Cyth1	cytohesin 1	116691	ENSRNOG00000043381
Egfr	NA	Egfr	epidermal growth factor receptor	24329	ENSRNOG00000004332
Ptk2	NA	Ptk2	PTK2 protein tyrosine kinase 2	25614	ENSRNOG00000007916
Nrg1	NA	Nrg1	neuregulin 1	112400	ENSRNOG00000010392
Plau	NA	Plau	plasminogen activator, urokinase	25619	ENSRNOG00000010516
Vcl	NA	Vcl	vinculin	305679	ENSRNOG00000010765
Cdh19	NA	Cdh19	cadherin 19, type 2	360835	ENSRNOG00000029841

biological process	cellular monovalent inorganic anion homeostasis	GO:0030320			
C=6;O=2;E=0.02;R=90.48;rawP=0.0002;adjP=0.0068					
Cacna1a	NA	Cacna1a	calcium channel, voltage-dependent, P/Q type, alpha 1A subunit	25398	ENSRNOG00000002559
Ptk2	NA	Ptk2	PTK2 protein tyrosine kinase 2	25614	ENSRNOG00000007916

biological process	cell adhesion	GO:0007155			
C=692;O=10;E=2.55;R=3.92;rawP=0.0002;adjP=0.0068					
Stab2	NA	Stab2	stabilin 2	282580	NULL
Cdh2	NA	Cdh2	cadherin 2	83501	ENSRNOG00000015602
Ephb1	NA	Ephb1	Eph receptor B1	24338	ENSRNOG00000007865
Cyth1	NA	Cyth1	cytohesin 1	116691	ENSRNOG00000043381
Egfr	NA	Egfr	epidermal growth factor receptor	24329	ENSRNOG00000004332
Ptk2	NA	Ptk2	PTK2 protein tyrosine kinase 2	25614	ENSRNOG00000007916
Nrg1	NA	Nrg1	neuregulin 1	112400	ENSRNOG00000010392
Plau	NA	Plau	plasminogen activator, urokinase	25619	ENSRNOG00000010516
Vcl	NA	Vcl	vinculin	305679	ENSRNOG00000010765
Cdh19	NA	Cdh19	cadherin 19, type 2	360835	ENSRNOG00000029841

biological process	cellular component movement	GO:0006928			
C=990;O=12;E=3.65;R=3.29;rawP=0.0002;adjP=0.0068					
Cdh2	NA	Cdh2	cadherin 2	83501	ENSRNOG00000015602
Ddr2	NA	Ddr2	discoidin domain receptor tyrosine kinase 2	685781	ENSRNOG00000002881
Ephb1	NA	Ephb1	Eph receptor B1	24338	ENSRNOG00000007865
Epha4	NA	Epha4	Eph receptor A4	316539	ENSRNOG00000013213
Myo9b	NA	Myo9b	myosin IXb	25486	ENSRNOG00000016256
Elmo1	NA	Elmo1	engulfment and cell motility 1	361251	ENSRNOG00000018726
Egfr	NA	Egfr	epidermal growth factor receptor	24329	ENSRNOG00000004332

Plau	NA	Plau	plasminogen activator, urokinase	25619	ENSRNOG00000010516
Ptk2	NA	Ptk2	PTK2 protein tyrosine kinase 2	25614	ENSRNOG00000007916
Nrg1	NA	Nrg1	neuregulin 1	112400	ENSRNOG00000010392
Dcdc2	NA	Dcdc2	doublecortin domain containing 2	291130	ENSRNOG00000017511
Vcl	NA	Vcl	vinculin	305679	ENSRNOG00000010765
biological process	multicellular organismal signaling	GO:0035637			
C=596;O=9;E=2.20;R=4.10;rawP=0.0003;adjP=0.0089					
Cacna1a	NA	Cacna1a	calcium channel, voltage-dependent, P/Q type, alpha 1A subunit	25398	ENSRNOG00000002559
Cdh2	NA	Cdh2	cadherin 2	83501	ENSRNOG00000015602
Ephb1	NA	Ephb1	Eph receptor B1	24338	ENSRNOG00000007865
Egfr	NA	Egfr	epidermal growth factor receptor	24329	ENSRNOG00000004332
Gria4	NA	Gria4	glutamate receptor, ionotropic, AMPA 4	29629	ENSRNOG00000006957
Ptk2	NA	Ptk2	PTK2 protein tyrosine kinase 2	25614	ENSRNOG00000007916
Nrg1	NA	Nrg1	neuregulin 1	112400	ENSRNOG00000010392
Camk2g	NA	Camk2g	calcium/calmodulin-dependent protein kinase II gamma	171140	ENSRNOG00000009783
Rheb	NA	Rheb	Ras homolog enriched in brain	26954	NULL
biological process	synaptic transmission	GO:0007268			
C=470;O=8;E=1.73;R=4.62;rawP=0.0003;adjP=0.0089					
Cacna1a	NA	Cacna1a	calcium channel, voltage-dependent, P/Q type, alpha 1A subunit	25398	ENSRNOG00000002559
Ephb1	NA	Ephb1	Eph receptor B1	24338	ENSRNOG00000007865
Egfr	NA	Egfr	epidermal growth factor receptor	24329	ENSRNOG00000004332
Gria4	NA	Gria4	glutamate receptor, ionotropic, AMPA 4	29629	ENSRNOG00000006957
Ptk2	NA	Ptk2	PTK2 protein tyrosine kinase 2	25614	ENSRNOG00000007916
Nrg1	NA	Nrg1	neuregulin 1	112400	ENSRNOG00000010392
Camk2g	NA	Camk2g	calcium/calmodulin-dependent protein kinase II gamma	171140	ENSRNOG00000009783
Rheb	NA	Rheb	Ras homolog enriched in brain	26954	NULL
biological process	cell-cell signaling	GO:0007267			
C=746;O=10;E=2.75;R=3.64;rawP=0.0003;adjP=0.0089					
Cacna1a	NA	Cacna1a	calcium channel, voltage-dependent, P/Q type, alpha 1A subunit	25398	ENSRNOG00000002559
Ephb1	NA	Ephb1	Eph receptor B1	24338	ENSRNOG00000007865
Ppp3cb	NA	Ppp3cb	protein phosphatase 3, catalytic subunit, beta isozyme	24675	ENSRNOG00000007757
Egfr	NA	Egfr	epidermal growth factor receptor	24329	ENSRNOG00000004332
Gria4	NA	Gria4	glutamate receptor, ionotropic, AMPA 4	29629	ENSRNOG00000006957

Ptk2	NA	Ptk2	PTK2 protein tyrosine kinase 2	25614	ENSRNOG00000007916
Nrg1	NA	Nrg1	neuregulin 1	112400	ENSRNOG00000010392
Camk2g	NA	Camk2g	calcium/calmodulin-dependent protein kinase II gamma	171140	ENSRNOG00000009783
Rheb	NA	Rheb	Ras homolog enriched in brain	26954	NULL
Sall1	NA	Sall1	sal-like 1 (Drosophila)	307740	NULL

biological process	cell projection morphogenesis	GO:0048858			
C=496;O=8;E=1.83;R=4.38;rawP=0.0004;adjP=0.0105					
Cacna1a	NA	Cacna1a	calcium channel, voltage-dependent, P/Q type, alpha 1A subunit	25398	ENSRNOG00000002559
Cdh2	NA	Cdh2	cadherin 2	83501	ENSRNOG00000015602
Ephb1	NA	Ephb1	Eph receptor B1	24338	ENSRNOG00000007865
Epha4	NA	Epha4	Eph receptor A4	316539	ENSRNOG00000013213
Myo9b	NA	Myo9b	myosin IXb	25486	ENSRNOG00000016256
Egfr	NA	Egfr	epidermal growth factor receptor	24329	ENSRNOG00000004332
Ptk2	NA	Ptk2	PTK2 protein tyrosine kinase 2	25614	ENSRNOG00000007916
Dcdc2	NA	Dcdc2	doublecortin domain containing 2	291130	ENSRNOG00000017511

biological process	cell morphogenesis	GO:0000902			
C=756;O=10;E=2.79;R=3.59;rawP=0.0004;adjP=0.0105					
Cacna1a	NA	Cacna1a	calcium channel, voltage-dependent, P/Q type, alpha 1A subunit	25398	ENSRNOG00000002559
Cdh2	NA	Cdh2	cadherin 2	83501	ENSRNOG00000015602
Ephb1	NA	Ephb1	Eph receptor B1	24338	ENSRNOG00000007865
Epha4	NA	Epha4	Eph receptor A4	316539	ENSRNOG00000013213
Myo9b	NA	Myo9b	myosin IXb	25486	ENSRNOG00000016256
Egfr	NA	Egfr	epidermal growth factor receptor	24329	ENSRNOG00000004332
Ptk2	NA	Ptk2	PTK2 protein tyrosine kinase 2	25614	ENSRNOG00000007916
Nrg1	NA	Nrg1	neuregulin 1	112400	ENSRNOG00000010392
Dcdc2	NA	Dcdc2	doublecortin domain containing 2	291130	ENSRNOG00000017511
Sall1	NA	Sall1	sal-like 1 (Drosophila)	307740	NULL

biological process	establishment or maintenance of cell polarity	GO:0007163			
C=97;O=4;E=0.36;R=11.19;rawP=0.0004;adjP=0.0105					
Sdccag8	NA	Sdccag8	serologically defined colon cancer antigen 8	305002	ENSRNOG00000004181
Ephb1	NA	Ephb1	Eph receptor B1	24338	ENSRNOG00000007865
Cyth1	NA	Cyth1	cytohesin 1	116691	ENSRNOG00000043381
Myo9b	NA	Myo9b	myosin IXb	25486	ENSRNOG00000016256

biological process	cell part morphogenesis	GO:0032990			
C=509;O=8;E=1.88;R=4.27;rawP=0.0005;adjP=0.0114					
Cacna1a	NA	Cacna1a	calcium channel, voltage-dependent, P/Q type, alpha 1A subunit	25398	ENSRNOG00000002559
Cdh2	NA	Cdh2	cadherin 2	83501	ENSRNOG00000015602
Ephb1	NA	Ephb1	Eph receptor B1	24338	ENSRNOG00000007865
Epha4	NA	Epha4	Eph receptor A4	316539	ENSRNOG00000013213
Myo9b	NA	Myo9b	myosin IXb	25486	ENSRNOG00000016256
Egfr	NA	Egfr	epidermal growth factor receptor	24329	ENSRNOG00000004332
Ptk2	NA	Ptk2	PTK2 protein tyrosine kinase 2	25614	ENSRNOG00000007916
Dcdc2	NA	Dcdc2	doublecortin domain containing 2	291130	ENSRNOG00000017511
biological process	locomotion	GO:0040011			
C=937;O=11;E=3.45;R=3.19;rawP=0.0005;adjP=0.0114					
Cdh2	NA	Cdh2	cadherin 2	83501	ENSRNOG00000015602
Ddr2	NA	Ddr2	discoidin domain receptor tyrosine kinase 2	685781	ENSRNOG00000002881
Ephb1	NA	Ephb1	Eph receptor B1	24338	ENSRNOG00000007865
Epha4	NA	Epha4	Eph receptor A4	316539	ENSRNOG00000013213
Myo9b	NA	Myo9b	myosin IXb	25486	ENSRNOG00000016256
Egfr	NA	Egfr	epidermal growth factor receptor	24329	ENSRNOG00000004332
Ptk2	NA	Ptk2	PTK2 protein tyrosine kinase 2	25614	ENSRNOG00000007916
Nrg1	NA	Nrg1	neuregulin 1	112400	ENSRNOG00000010392
Plau	NA	Plau	plasminogen activator, urokinase	25619	ENSRNOG00000010516
Dcdc2	NA	Dcdc2	doublecortin domain containing 2	291130	ENSRNOG00000017511
Vcl	NA	Vcl	vinculin	305679	ENSRNOG00000010765
biological process	regulation of axonogenesis	GO:0050770			
C=101;O=4;E=0.37;R=10.75;rawP=0.0005;adjP=0.0114					
Cacna1a	NA	Cacna1a	calcium channel, voltage-dependent, P/Q type, alpha 1A subunit	25398	ENSRNOG00000002559
Cdh2	NA	Cdh2	cadherin 2	83501	ENSRNOG00000015602
Epha4	NA	Epha4	Eph receptor A4	316539	ENSRNOG00000013213
Ptk2	NA	Ptk2	PTK2 protein tyrosine kinase 2	25614	ENSRNOG00000007916
biological process	regulation of signaling	GO:0023051			
C=1791;O=16;E=6.60;R=2.42;rawP=0.0005;adjP=0.0114					

Cacna1a	NA	Cacna1a	calcium channel, voltage-dependent, P/Q type, alpha 1A subunit	25398	ENSRNOG00000002559
Cdh2	NA	Cdh2	cadherin 2	83501	ENSRNOG00000015602
Ephb1	NA	Ephb1	Eph receptor B1	24338	ENSRNOG00000007865
Myo9b	NA	Myo9b	myosin IXb	25486	ENSRNOG00000016256
Gria4	NA	Gria4	glutamate receptor, ionotropic, AMPA 4	29629	ENSRNOG00000006957
Camk2g	NA	Camk2g	calcium/calmodulin-dependent protein kinase II gamma	171140	ENSRNOG00000009783
Plau	NA	Plau	plasminogen activator, urokinase	25619	ENSRNOG00000010516
Sall1	NA	Sall1	sal-like 1 (Drosophila)	307740	NULL
Cyth1	NA	Cyth1	cytohesin 1	116691	ENSRNOG00000043381
Epha4	NA	Epha4	Eph receptor A4	316539	ENSRNOG00000013213
Ppp3cb	NA	Ppp3cb	protein phosphatase 3, catalytic subunit, beta isozyme	24675	ENSRNOG00000007757
Egfr	NA	Egfr	epidermal growth factor receptor	24329	ENSRNOG00000004332
Nrg1	NA	Nrg1	neuregulin 1	112400	ENSRNOG00000010392
Ptk2	NA	Ptk2	PTK2 protein tyrosine kinase 2	25614	ENSRNOG00000007916
Rheb	NA	Rheb	Ras homolog enriched in brain	26954	NULL
Uaca	NA	Uaca	uveal autoantigen with coiled-coil domains and ankyrin repeats	315732	NULL

biological process	neuron projection morphogenesis	GO:0048812			
C=396;O=7;E=1.46;R=4.80;rawP=0.0006;adjP=0.0129					
Cacna1a	NA	Cacna1a	calcium channel, voltage-dependent, P/Q type, alpha 1A subunit	25398	ENSRNOG00000002559
Egfr	NA	Egfr	epidermal growth factor receptor	24329	ENSRNOG00000004332
Cdh2	NA	Cdh2	cadherin 2	83501	ENSRNOG00000015602
Ephb1	NA	Ephb1	Eph receptor B1	24338	ENSRNOG00000007865
Epha4	NA	Epha4	Eph receptor A4	316539	ENSRNOG00000013213
Ptk2	NA	Ptk2	PTK2 protein tyrosine kinase 2	25614	ENSRNOG00000007916
Dcdc2	NA	Dcdc2	doublecortin domain containing 2	291130	ENSRNOG00000017511

biological process	localization	GO:0051179			
C=3501;O=24;E=12.90;R=1.86;rawP=0.0006;adjP=0.0129					
Cacna1a	NA	Cacna1a	calcium channel, voltage-dependent, P/Q type, alpha 1A subunit	25398	ENSRNOG00000002559
Cdh2	NA	Cdh2	cadherin 2	83501	ENSRNOG00000015602
Ddr2	NA	Ddr2	discoidin domain receptor tyrosine kinase 2	685781	ENSRNOG00000002881
Ephb1	NA	Ephb1	Eph receptor B1	24338	ENSRNOG00000007865
Sec24c	NA	Sec24c	SEC24 family, member C (S. cerevisiae)	685144	ENSRNOG00000009042
Brca2	NA	Brca2	breast cancer 2	360254	ENSRNOG00000001111
Myo9b	NA	Myo9b	myosin IXb	25486	ENSRNOG00000016256
Elmo1	NA	Elmo1	engulfment and cell motility 1	361251	ENSRNOG00000018726
Camk2g	NA	Camk2g	calcium/calmodulin-dependent protein kinase II gamma	171140	ENSRNOG00000009783

Plau	NA	Plau	plasminogen activator, urokinase	25619	ENSRNOG00000010516
Dcdc2	NA	Dcdc2	doublecortin domain containing 2	291130	ENSRNOG00000017511
Vcl	NA	Vcl	vinculin	305679	ENSRNOG00000010765
Tbc1d1	NA	Tbc1d1	TBC1 (tre-2/USP6, BUB2, cdc16) domain family, member 1	360937	ENSRNOG00000002180
Kcnt2	NA	Kcnt2	potassium channel, subfamily T, member 2	304827	ENSRNOG00000013312
Stab2	NA	Stab2	stabilin 2	282580	NULL
Itln1	NA	Itln1	intelectin 1 (galactofuranose binding)	498284	ENSRNOG00000004678
Ppp3cb	NA	Ppp3cb	protein phosphatase 3, catalytic subunit, beta isozyme	24675	ENSRNOG00000007757
Epha4	NA	Epha4	Eph receptor A4	316539	ENSRNOG00000013213
Cyth1	NA	Cyth1	cytohesin 1	116691	ENSRNOG00000043381
Ccdc91	NA	Ccdc91	coiled-coil domain containing 91	312863	NULL
Egfr	NA	Egfr	epidermal growth factor receptor	24329	ENSRNOG00000004332
Nrg1	NA	Nrg1	neuregulin 1	112400	ENSRNOG00000010392
Ptk2	NA	Ptk2	PTK2 protein tyrosine kinase 2	25614	ENSRNOG00000007916
Uaca	NA	Uaca	uveal autoantigen with coiled-coil domains and ankyrin repeats	315732	NULL

biological process	Ras protein signal transduction	GO:0007265			
C=292;O=6;E=1.08;R=5.58;rawP=0.0007;adjP=0.0142					
Elmo1	NA	Elmo1	engulfment and cell motility 1	361251	ENSRNOG00000018726
Cdh2	NA	Cdh2	cadherin 2	83501	ENSRNOG00000015602
Epha4	NA	Epha4	Eph receptor A4	316539	ENSRNOG00000013213
Nrg1	NA	Nrg1	neuregulin 1	112400	ENSRNOG00000010392
Cyth1	NA	Cyth1	cytohesin 1	116691	ENSRNOG00000043381
Myo9b	NA	Myo9b	myosin IXb	25486	ENSRNOG00000016256

biological process	cellular component morphogenesis	GO:0032989			
C=820;O=10;E=3.02;R=3.31;rawP=0.0007;adjP=0.0142					
Cacna1a	NA	Cacna1a	calcium channel, voltage-dependent, P/Q type, alpha 1A subunit	25398	ENSRNOG00000002559
Cdh2	NA	Cdh2	cadherin 2	83501	ENSRNOG00000015602
Ephb1	NA	Ephb1	Eph receptor B1	24338	ENSRNOG00000007865
Epha4	NA	Epha4	Eph receptor A4	316539	ENSRNOG00000013213
Myo9b	NA	Myo9b	myosin IXb	25486	ENSRNOG00000016256
Egfr	NA	Egfr	epidermal growth factor receptor	24329	ENSRNOG00000004332
Ptk2	NA	Ptk2	PTK2 protein tyrosine kinase 2	25614	ENSRNOG00000007916
Nrg1	NA	Nrg1	neuregulin 1	112400	ENSRNOG00000010392
Dcdc2	NA	Dcdc2	doublecortin domain containing 2	291130	ENSRNOG00000017511
Sall1	NA	Sall1	sal-like 1 (Drosophila)	307740	NULL

biological process	cellular anion homeostasis	GO:0030002				
C=12;O=2;E=0.04;R=45.24;rawP=0.0009;adjP=0.0177						
Cacna1a	NA	Cacna1a	calcium channel, voltage-dependent, P/Q type, alpha 1A subunit	25398	ENSRNOG00000002559
Ptk2	NA	Ptk2	PTK2 protein tyrosine kinase 2	25614	ENSRNOG00000007916
biological process	peptidyl-tyrosine modification	GO:0018212			
C=211;O=5;E=0.78;R=6.43;rawP=0.0010;adjP=0.0187					
Egfr	NA	Egfr	epidermal growth factor receptor	24329	ENSRNOG00000004332
Ddr2	NA	Ddr2	discoidin domain receptor tyrosine kinase 2	685781	ENSRNOG00000002881
Epha4	NA	Epha4	Eph receptor A4	316539	ENSRNOG00000013213
Nrg1	NA	Nrg1	neuregulin 1	112400	ENSRNOG00000010392
Ptk2	NA	Ptk2	PTK2 protein tyrosine kinase 2	25614	ENSRNOG00000007916
biological process	peptidyl-tyrosine phosphorylation	GO:0018108			
C=209;O=5;E=0.77;R=6.49;rawP=0.0010;adjP=0.0187					
Egfr	NA	Egfr	epidermal growth factor receptor	24329	ENSRNOG00000004332
Ddr2	NA	Ddr2	discoidin domain receptor tyrosine kinase 2	685781	ENSRNOG00000002881
Epha4	NA	Epha4	Eph receptor A4	316539	ENSRNOG00000013213
Nrg1	NA	Nrg1	neuregulin 1	112400	ENSRNOG00000010392
Ptk2	NA	Ptk2	PTK2 protein tyrosine kinase 2	25614	ENSRNOG00000007916
biological process	regulation of catabolic process	GO:0009894			
C=442;O=7;E=1.63;R=4.30;rawP=0.0011;adjP=0.0195					
Egfr	NA	Egfr	epidermal growth factor receptor	24329	ENSRNOG00000004332
Epha4	NA	Epha4	Eph receptor A4	316539	ENSRNOG00000013213
Nrg1	NA	Nrg1	neuregulin 1	112400	ENSRNOG00000010392
Ptk2	NA	Ptk2	PTK2 protein tyrosine kinase 2	25614	ENSRNOG00000007916
Myo9b	NA	Myo9b	myosin IXb	25486	ENSRNOG00000016256
Uaca	NA	Uaca	uveal autoantigen with coiled-coil domains and ankyrin repeats	315732	NULL
Scoc	NA	Scoc	short coiled-coil protein	364981	ENSRNOG00000003853
biological process	neuron differentiation	GO:0030182			

C=868;O=10;E=3.20;R=3.13;rawP=0.0011;adjP=0.0195

Cacna1a	NA	Cacna1a	calcium channel, voltage-dependent, P/Q type, alpha 1A subunit	25398	ENSRNOG00000002559
Cdh2	NA	Cdh2	cadherin 2	83501	ENSRNOG00000015602
Ephb1	NA	Ephb1	Eph receptor B1	24338	ENSRNOG00000007865
Epha4	NA	Epha4	Eph receptor A4	316539	ENSRNOG00000013213
Egfr	NA	Egfr	epidermal growth factor receptor	24329	ENSRNOG00000004332
Ptk2	NA	Ptk2	PTK2 protein tyrosine kinase 2	25614	ENSRNOG00000007916
Nrg1	NA	Nrg1	neuregulin 1	112400	ENSRNOG00000010392
Cdk5rap1	NA	Cdk5rap1	CDK5 regulatory subunit associated protein 1	252827	ENSRNOG00000015696
Dcdc2	NA	Dcdc2	doublecortin domain containing 2	291130	ENSRNOG00000017511
Sall1	NA	Sall1	sal-like 1 (Drosophila)	307740	NULL

molecular function transmembrane receptor protein tyrosine kinase activity GO:0004714

C=55;O=4;E=0.19;R=21.03;rawP=3.79e-05;adjP=0.0021

Egfr	NA	Egfr	epidermal growth factor receptor	24329	ENSRNOG00000004332
Ddr2	NA	Ddr2	discoidin domain receptor tyrosine kinase 2	685781	ENSRNOG00000002881
Ephb1	NA	Ephb1	Eph receptor B1	24338	ENSRNOG00000007865
Epha4	NA	Epha4	Eph receptor A4	316539	ENSRNOG00000013213

molecular function transferase activity GO:0016740

C=1632;O=16;E=5.64;R=2.84;rawP=7.46e-05;adjP=0.0021

Fut11	NA	Fut11	fucosyltransferase 11 (alpha (1,3) fucosyltransferase)	286971	ENSRNOG00000009274
Ddr2	NA	Ddr2	discoidin domain receptor tyrosine kinase 2	685781	ENSRNOG00000002881
Ephb1	NA	Ephb1	Eph receptor B1	24338	ENSRNOG00000007865
Brca2	NA	Brca2	breast cancer 2	360254	ENSRNOG00000001111
LOC685793	NA	LOC685793	similar to serine/threonine kinase	685793	ENSRNOG00000030545
Ndst2	NA	Ndst2	N-deacetylase/N-sulfotransferase (heparan glucosaminyl) 2	114002	ENSRNOG00000027171
Camk2g	NA	Camk2g	calcium/calmodulin-dependent protein kinase II gamma	171140	ENSRNOG00000009783
Plau	NA	Plau	plasminogen activator, urokinase	25619	ENSRNOG00000010516
Epha4	NA	Epha4	Eph receptor A4	316539	ENSRNOG00000013213
Gtdc1	NA	Gtdc1	glycosyltransferase-like domain containing 1	362129	ENSRNOG00000031038
Cers4	NA	Cers4	ceramide synthase 4	304208	ENSRNOG00000001072
Egfr	NA	Egfr	epidermal growth factor receptor	24329	ENSRNOG00000004332
RGD1310572	NA	RGD1310572	similar to calmegin	304646	ENSRNOG00000003695
Ptk2	NA	Ptk2	PTK2 protein tyrosine kinase 2	25614	ENSRNOG00000007916
Cdk5rap1	NA	Cdk5rap1	CDK5 regulatory subunit associated protein 1	252827	ENSRNOG00000015696
Galk2	NA	Galk2	galactokinase 2	296117	ENSRNOG00000009289

molecular function	protein tyrosine kinase activity	GO:0004713			
C=120;O=5;E=0.41;R=12.05;rawP=5.69e-05;adjP=0.0021					
Egfr	NA	Egfr	epidermal growth factor receptor	24329	ENSRNOG00000004332
Ddr2	NA	Ddr2	discoidin domain receptor tyrosine kinase 2	685781	ENSRNOG00000002881
Ephb1	NA	Ephb1	Eph receptor B1	24338	ENSRNOG00000007865
Epha4	NA	Epha4	Eph receptor A4	316539	ENSRNOG00000013213
Ptk2	NA	Ptk2	PTK2 protein tyrosine kinase 2	25614	ENSRNOG00000007916
molecular function	transmembrane-ephrin receptor activity	GO:0005005			
C=4;O=2;E=0.01;R=144.61;rawP=7.00e-05;adjP=0.0021					
Ephb1	NA	Ephb1	Eph receptor B1	24338	ENSRNOG00000007865
Epha4	NA	Epha4	Eph receptor A4	316539	ENSRNOG00000013213
molecular function	transmembrane receptor protein kinase activity	GO:0019199			
C=70;O=4;E=0.24;R=16.53;rawP=9.80e-05;adjP=0.0022					
Egfr	NA	Egfr	epidermal growth factor receptor	24329	ENSRNOG00000004332
Ddr2	NA	Ddr2	discoidin domain receptor tyrosine kinase 2	685781	ENSRNOG00000002881
Ephb1	NA	Ephb1	Eph receptor B1	24338	ENSRNOG00000007865
Epha4	NA	Epha4	Eph receptor A4	316539	ENSRNOG00000013213
molecular function	alpha-catenin binding	GO:0045294			
C=7;O=2;E=0.02;R=82.63;rawP=0.0002;adjP=0.0038					
Cdh2	NA	Cdh2	cadherin 2	83501	ENSRNOG00000015602
Vcl	NA	Vcl	vinculin	305679	ENSRNOG00000010765
molecular function	calmodulin binding	GO:0005516			
C=117;O=4;E=0.40;R=9.89;rawP=0.0007;adjP=0.0114					
Cacna1a	NA	Cacna1a	calcium channel, voltage-dependent, P/Q type, alpha 1A subunit	25398	ENSRNOG00000002559
Ppp3cb	NA	Ppp3cb	protein phosphatase 3, catalytic subunit, beta isozyme	24675	ENSRNOG00000007757
Camk2g	NA	Camk2g	calcium/calmodulin-dependent protein kinase II gamma	171140	ENSRNOG00000009783
Myo9b	NA	Myo9b	myosin IXb	25486	ENSRNOG00000016256

molecular function	kinase activity	GO:0016301				
C=754;O=9;E=2.61;R=3.45;rawP=0.0010;adjP=0.0127						
Ddr2	NA	Ddr2	discoidin domain receptor tyrosine kinase 2	685781	ENSRNOG00000002881
Ephb1	NA	Ephb1	Eph receptor B1	24338	ENSRNOG00000007865
Epha4	NA	Epha4	Eph receptor A4	316539	ENSRNOG00000013213
LOC685793	NA	LOC685793	similar to serine/threonine kinase	685793	ENSRNOG00000030545
Egfr	NA	Egfr	epidermal growth factor receptor	24329	ENSRNOG00000004332
Ptk2	NA	Ptk2	PTK2 protein tyrosine kinase 2	25614	ENSRNOG00000007916
Plau	NA	Plau	plasminogen activator, urokinase	25619	ENSRNOG00000010516
Camk2g	NA	Camk2g	calcium/calmodulin-dependent protein kinase II gamma	171140	ENSRNOG00000009783
Galk2	NA	Galk2	galactokinase 2	296117	ENSRNOG00000009289

molecular function	ephrin receptor activity	GO:0005003			
C=13;O=2;E=0.04;R=44.50;rawP=0.0009;adjP=0.0127					
Ephb1	NA	Ephb1	Eph receptor B1	24338	ENSRNOG00000007865
Epha4	NA	Epha4	Eph receptor A4	316539	ENSRNOG00000013213

molecular function	phosphotransferase activity, alcohol group as acceptor	GO:0016773			
C=688;O=8;E=2.38;R=3.36;rawP=0.0023;adjP=0.0237					
Ddr2	NA	Ddr2	discoidin domain receptor tyrosine kinase 2	685781	ENSRNOG00000002881
Ephb1	NA	Ephb1	Eph receptor B1	24338	ENSRNOG00000007865
Epha4	NA	Epha4	Eph receptor A4	316539	ENSRNOG00000013213
LOC685793	NA	LOC685793	similar to serine/threonine kinase	685793	ENSRNOG00000030545
Egfr	NA	Egfr	epidermal growth factor receptor	24329	ENSRNOG00000004332
Ptk2	NA	Ptk2	PTK2 protein tyrosine kinase 2	25614	ENSRNOG00000007916
Camk2g	NA	Camk2g	calcium/calmodulin-dependent protein kinase II gamma	171140	ENSRNOG00000009783
Galk2	NA	Galk2	galactokinase 2	296117	ENSRNOG00000009289

molecular function	transferase activity, transferring phosphorus-containing groups	GO:0016772			
C=871;O=9;E=3.01;R=2.99;rawP=0.0027;adjP=0.0237					
Ddr2	NA	Ddr2	discoidin domain receptor tyrosine kinase 2	685781	ENSRNOG00000002881
Ephb1	NA	Ephb1	Eph receptor B1	24338	ENSRNOG00000007865
Epha4	NA	Epha4	Eph receptor A4	316539	ENSRNOG00000013213
LOC685793	NA	LOC685793	similar to serine/threonine kinase	685793	ENSRNOG00000030545
Egfr	NA	Egfr	epidermal growth factor receptor	24329	ENSRNOG00000004332

Ptk2	NA	Ptk2	PTK2 protein tyrosine kinase 2	25614	ENSRNOG00000007916
Plau	NA	Plau	plasminogen activator, urokinase	25619	ENSRNOG00000010516
Camk2g	NA	Camk2g	calcium/calmodulin-dependent protein kinase II gamma	171140	ENSRNOG00000009783
Galk2	NA	Galk2	galactokinase 2	296117	ENSRNOG00000009289
molecular function	Ras GTPase activator activity	GO:0005099			
C=78;O=3;E=0.27;R=11.12;rawP=0.0025;adjP=0.0237					
Tbc1d1	NA	Tbc1d1	TBC1 (tre-2/USP6, BUB2, cdc16) domain family, member 1	360937	ENSRNOG00000002180
Myo9b	NA	Myo9b	myosin IXb	25486	ENSRNOG00000016256
Tbc1d5	NA	Tbc1d5	TBC1 domain family, member 5	501088	ENSRNOG00000010637
molecular function	protein phosphatase binding	GO:0019903			
C=81;O=3;E=0.28;R=10.71;rawP=0.0027;adjP=0.0237					
Egfr	NA	Egfr	epidermal growth factor receptor	24329	ENSRNOG00000004332
Cdh2	NA	Cdh2	cadherin 2	83501	ENSRNOG00000015602
Ppp3cb	NA	Ppp3cb	protein phosphatase 3, catalytic subunit, beta isozyme	24675	ENSRNOG00000007757
molecular function	protein kinase activity	GO:0004672			
C=583;O=7;E=2.02;R=3.47;rawP=0.0037;adjP=0.0301					
Egfr	NA	Egfr	epidermal growth factor receptor	24329	ENSRNOG00000004332
Ddr2	NA	Ddr2	discoidin domain receptor tyrosine kinase 2	685781	ENSRNOG00000002881
Ephb1	NA	Ephb1	Eph receptor B1	24338	ENSRNOG00000007865
Epha4	NA	Epha4	Eph receptor A4	316539	ENSRNOG00000013213
Camk2g	NA	Camk2g	calcium/calmodulin-dependent protein kinase II gamma	171140	ENSRNOG00000009783
Ptk2	NA	Ptk2	PTK2 protein tyrosine kinase 2	25614	ENSRNOG00000007916
LOC685793	NA	LOC685793	similar to serine/threonine kinase	685793	ENSRNOG00000030545
molecular function	enzyme regulator activity	GO:0030234			
C=787;O=8;E=2.72;R=2.94;rawP=0.0052;adjP=0.0395					
Cyth1	NA	Cyth1	cytohesin 1	116691	ENSRNOG00000043381
Myo9b	NA	Myo9b	myosin IXb	25486	ENSRNOG00000016256
Tbc1d5	NA	Tbc1d5	TBC1 domain family, member 5	501088	ENSRNOG00000010637
Egfr	NA	Egfr	epidermal growth factor receptor	24329	ENSRNOG00000004332
Nrg1	NA	Nrg1	neuregulin 1	112400	ENSRNOG00000010392
Cdk5rap1	NA	Cdk5rap1	CDK5 regulatory subunit associated protein 1	252827	ENSRNOG00000015696

Mup5	NA	Mup5	major urinary protein 5	298107	ENSRNOG00000033130
Tbc1d1	NA	Tbc1d1	TBC1 (tre-2/USP6, BUB2, cdc16) domain family, member 1	360937	ENSRNOG00000002180

molecular function	small GTPase regulator activity	GO:0005083			
C=219;O=4;E=0.76;R=5.28;rawP=0.0068;adjP=0.0456					
Cyth1	NA	Cyth1	cytohesin 1	116691	ENSRNOG00000043381
Tbc1d1	NA	Tbc1d1	TBC1 (tre-2/USP6, BUB2, cdc16) domain family, member 1	360937	ENSRNOG00000002180
Myo9b	NA	Myo9b	myosin IXb	25486	ENSRNOG00000016256
Tbc1d5	NA	Tbc1d5	TBC1 domain family, member 5	501088	ENSRNOG00000010637

molecular function	Rab GTPase activator activity	GO:0005097			
C=35;O=2;E=0.12;R=16.53;rawP=0.0065;adjP=0.0456					
Tbc1d1	NA	Tbc1d1	TBC1 (tre-2/USP6, BUB2, cdc16) domain family, member 1	360937	ENSRNOG00000002180
Tbc1d5	NA	Tbc1d5	TBC1 domain family, member 5	501088	ENSRNOG00000010637

cellular component	cell part	GO:0044464			
C=11952;O=52;E=42.66;R=1.22;rawP=0.0006;adjP=0.0198					
Cacna1a	NA	Cacna1a	calcium channel, voltage-dependent, P/Q type, alpha 1A subunit	25398	ENSRNOG00000002559
Ephb1	NA	Ephb1	Eph receptor B1	24338	ENSRNOG00000007865
Elmo1	NA	Elmo1	engulfment and cell motility 1	361251	ENSRNOG00000018726
Ndst2	NA	Ndst2	N-deacetylase/N-sulfotransferase (heparan glucosaminyl) 2	114002	ENSRNOG00000027171
Gria4	NA	Gria4	glutamate receptor, ionotropic, AMPA 4	29629	ENSRNOG00000006957
Hmbox1	NA	Hmbox1	homeobox containing 1	305968	ENSRNOG00000013326
Clgn	NA	Clgn	calmegin	685504	ENSRNOG00000003755
Cyth1	NA	Cyth1	cytohesin 1	116691	ENSRNOG00000043381
Scoc	NA	Scoc	short coiled-coil protein	364981	ENSRNOG00000003853
Glcci1	NA	Glcci1	glucocorticoid induced transcript 1	500026	NULL
Egfr	NA	Egfr	epidermal growth factor receptor	24329	ENSRNOG00000004332
Hormad2	NA	Hormad2	HORMA domain containing 2	498400	ENSRNOG00000037865
Ptk2	NA	Ptk2	PTK2 protein tyrosine kinase 2	25614	ENSRNOG00000007916
Nrg1	NA	Nrg1	neuregulin 1	112400	ENSRNOG00000010392
Galk2	NA	Galk2	galactokinase 2	296117	ENSRNOG00000009289
Uaca	NA	Uaca	uveal autoantigen with coiled-coil domains and ankyrin repeats	315732	NULL
Fut11	NA	Fut11	fucosyltransferase 11 (alpha (1,3) fucosyltransferase)	286971	ENSRNOG00000009274
Ddr2	NA	Ddr2	discoidin domain receptor tyrosine kinase 2	685781	ENSRNOG00000002881
Cdh2	NA	Cdh2	cadherin 2	83501	ENSRNOG00000015602

Emr4	NA	Emr4	EGF-like module containing, mucin-like, hormone receptor-like sequence 4	450235	NULL
Ccdc15	NA	Ccdc15	coiled-coil domain containing 15	367056	ENSRNOG00000032180
Brca2	NA	Brca2	breast cancer 2	360254	ENSRNOG00000001111
Sec24c	NA	Sec24c	SEC24 family, member C (S. cerevisiae)	685144	ENSRNOG00000009042
Myo9b	NA	Myo9b	myosin IXb	25486	ENSRNOG00000016256
Tbc1d5	NA	Tbc1d5	TBC1 domain family, member 5	501088	ENSRNOG00000010637
Sdccag8	NA	Sdccag8	serologically defined colon cancer antigen 8	305002	ENSRNOG00000004181
Arid2	NA	Arid2	AT rich interactive domain 2 (Arid-rfx like)	366980	ENSRNOG00000004831
Plau	NA	Plau	plasminogen activator, urokinase	25619	ENSRNOG00000010516
Camk2g	NA	Camk2g	calcium/calmodulin-dependent protein kinase II gamma	171140	ENSRNOG00000009783
Dcdc2	NA	Dcdc2	doublecortin domain containing 2	291130	ENSRNOG00000017511
Chchd1	NA	Chchd1	coiled-coil-helix-coiled-coil-helix domain containing 1	361005	ENSRNOG00000009297
Vcl	NA	Vcl	vinculin	305679	ENSRNOG00000010765
Tbc1d1	NA	Tbc1d1	TBC1 (tre-2/USP6, BUB2, cdc16) domain family, member 1	360937	ENSRNOG00000002180
Kcnt2	NA	Kcnt2	potassium channel, subfamily T, member 2	304827	ENSRNOG00000013312
Kdm4c	NA	Kdm4c	lysine (K)-specific demethylase 4C	298144	ENSRNOG00000006644
Sall1	NA	Sall1	sal-like 1 (Drosophila)	307740	NULL
Cdh19	NA	Cdh19	cadherin 19, type 2	360835	ENSRNOG00000029841
Stab2	NA	Stab2	stabilin 2	282580	NULL
Itln1	NA	Itln1	intelectin 1 (galactofuranose binding)	498284	ENSRNOG00000004678
Ppp3cb	NA	Ppp3cb	protein phosphatase 3, catalytic subunit, beta isozyme	24675	ENSRNOG00000007757
Pgm2	NA	Pgm2	phosphoglucomutase 2	289632	NULL
Epha4	NA	Epha4	Eph receptor A4	316539	ENSRNOG00000013213
Cers4	NA	Cers4	ceramide synthase 4	304208	ENSRNOG00000001072
Lpin2	NA	Lpin2	lipin 2	316737	ENSRNOG00000014876
Vom2r37	NA	Vom2r37	vomeronasal 2 receptor, 37	690356	ENSRNOG00000043280
Ccdc91	NA	Ccdc91	coiled-coil domain containing 91	312863	NULL
Ankrd32	NA	Ankrd32	ankyrin repeat domain 32	294601	ENSRNOG00000040279
Cdk5rap1	NA	Cdk5rap1	CDK5 regulatory subunit associated protein 1	252827	ENSRNOG00000015696
LOC688241	NA	LOC688241	similar to GTPase activating protein testicular GAP1	688241	NULL
Tecr	NA	Tecr	trans-2,3-enoyl-CoA reductase	191576	ENSRNOG00000021808
Cpne5	NA	Cpne5	copine V	309650	ENSRNOG00000000522
Rheb	NA	Rheb	Ras homolog enriched in brain	26954	NULL

cellular component	cell	GO:0005623			
C=11953;O=52;E=42.66;R=1.22;rawP=0.0007;adjP=0.0198					
Cacna1a	NA	Cacna1a	calcium channel, voltage-dependent, P/Q type, alpha 1A subunit	25398	ENSRNOG00000002559
Ephb1	NA	Ephb1	Eph receptor B1	24338	ENSRNOG00000007865
Elmo1	NA	Elmo1	engulfment and cell motility 1	361251	ENSRNOG00000018726

Ndst2	NA	Ndst2	N-deacetylase/N-sulfotransferase (heparan glucosaminyl) 2	114002	ENSRNOG00000027171
Gria4	NA	Gria4	glutamate receptor, ionotropic, AMPA 4	29629	ENSRNOG00000006957
Hmbox1	NA	Hmbox1	homeobox containing 1	305968	ENSRNOG00000013326
Clgn	NA	Clgn	calmegin	685504	ENSRNOG00000003755
Cyth1	NA	Cyth1	cytohesin 1	116691	ENSRNOG00000043381
Scoc	NA	Scoc	short coiled-coil protein	364981	ENSRNOG00000003853
Glcci1	NA	Glcci1	glucocorticoid induced transcript 1	500026	NULL
Egfr	NA	Egfr	epidermal growth factor receptor	24329	ENSRNOG00000004332
Hormad2	NA	Hormad2	HORMA domain containing 2	498400	ENSRNOG00000037865
Ptk2	NA	Ptk2	PTK2 protein tyrosine kinase 2	25614	ENSRNOG00000007916
Nrg1	NA	Nrg1	neuregulin 1	112400	ENSRNOG00000010392
Galk2	NA	Galk2	galactokinase 2	296117	ENSRNOG00000009289
Uaca	NA	Uaca	uveal autoantigen with coiled-coil domains and ankyrin repeats	315732	NULL
Fut11	NA	Fut11	fucosyltransferase 11 (alpha (1,3) fucosyltransferase)	286971	ENSRNOG00000009274
Ddr2	NA	Ddr2	discoidin domain receptor tyrosine kinase 2	685781	ENSRNOG00000002881
Cdh2	NA	Cdh2	cadherin 2	83501	ENSRNOG00000015602
Emr4	NA	Emr4	EGF-like module containing, mucin-like, hormone receptor-like sequence 4	450235	NULL
Ccdc15	NA	Ccdc15	coiled-coil domain containing 15	367056	ENSRNOG00000032180
Brca2	NA	Brca2	breast cancer 2	360254	ENSRNOG00000001111
Sec24c	NA	Sec24c	SEC24 family, member C (S. cerevisiae)	685144	ENSRNOG00000009042
Myo9b	NA	Myo9b	myosin IXb	25486	ENSRNOG00000016256
Tbc1d5	NA	Tbc1d5	TBC1 domain family, member 5	501088	ENSRNOG00000010637
Sdccag8	NA	Sdccag8	serologically defined colon cancer antigen 8	305002	ENSRNOG00000004181
Arid2	NA	Arid2	AT rich interactive domain 2 (Arid-rfx like)	366980	ENSRNOG00000004831
Plau	NA	Plau	plasminogen activator, urokinase	25619	ENSRNOG00000010516
Camk2g	NA	Camk2g	calcium/calmodulin-dependent protein kinase II gamma	171140	ENSRNOG00000009783
Dcdc2	NA	Dcdc2	doublecortin domain containing 2	291130	ENSRNOG00000017511
Chchd1	NA	Chchd1	coiled-coil-helix-coiled-coil-helix domain containing 1	361005	ENSRNOG00000009297
Vcl	NA	Vcl	vinculin	305679	ENSRNOG00000010765
Tbc1d1	NA	Tbc1d1	TBC1 (tre-2/USP6, BUB2, cdc16) domain family, member 1	360937	ENSRNOG00000002180
Kcnt2	NA	Kcnt2	potassium channel, subfamily T, member 2	304827	ENSRNOG00000013312
Kdm4c	NA	Kdm4c	lysine (K)-specific demethylase 4C	298144	ENSRNOG00000006644
Sall1	NA	Sall1	sal-like 1 (Drosophila)	307740	NULL
Cdh19	NA	Cdh19	cadherin 19, type 2	360835	ENSRNOG00000029841
Stab2	NA	Stab2	stabilin 2	282580	NULL
Itln1	NA	Itln1	intelectin 1 (galactofuranose binding)	498284	ENSRNOG00000004678
Ppp3cb	NA	Ppp3cb	protein phosphatase 3, catalytic subunit, beta isozyme	24675	ENSRNOG00000007757
Pgm2	NA	Pgm2	phosphoglucomutase 2	289632	NULL
Epha4	NA	Epha4	Eph receptor A4	316539	ENSRNOG00000013213
Cers4	NA	Cers4	ceramide synthase 4	304208	ENSRNOG00000001072
Lpin2	NA	Lpin2	lipin 2	316737	ENSRNOG00000014876

Vom2r37	NA	Vom2r37	vomeronasal 2 receptor, 37	690356	ENSRNOG00000043280
Ccdc91	NA	Ccdc91	coiled-coil domain containing 91	312863	NULL
Ankrd32	NA	Ankrd32	ankyrin repeat domain 32	294601	ENSRNOG00000040279
Cdk5rap1	NA	Cdk5rap1	CDK5 regulatory subunit associated protein 1	252827	ENSRNOG00000015696
LOC688241	NA	LOC688241	similar to GTPase activating protein testicular GAP1	688241	NULL
Tecr	NA	Tecr	trans-2,3-enoyl-CoA reductase	191576	ENSRNOG00000021808
Cpne5	NA	Cpne5	copine V	309650	ENSRNOG00000000522
Rheb	NA	Rheb	Ras homolog enriched in brain	26954	NULL
cellular component	intercalated disc	GO:0014704			
C=38;O=3;E=0.14;R=22.12;rawP=0.0003;adjP=0.0198					
Cdh2	NA	Cdh2	cadherin 2	83501	ENSRNOG00000015602
Ptk2	NA	Ptk2	PTK2 protein tyrosine kinase 2	25614	ENSRNOG00000007916
Vcl	NA	Vcl	vinculin	305679	ENSRNOG00000010765
cellular component	cell-cell contact zone	GO:0044291			
C=40;O=3;E=0.14;R=21.02;rawP=0.0004;adjP=0.0198					
Cdh2	NA	Cdh2	cadherin 2	83501	ENSRNOG00000015602
Ptk2	NA	Ptk2	PTK2 protein tyrosine kinase 2	25614	ENSRNOG00000007916
Vcl	NA	Vcl	vinculin	305679	ENSRNOG00000010765
cellular component	fascia adherens	GO:0005916			
C=13;O=2;E=0.05;R=43.11;rawP=0.0010;adjP=0.0226					
Cdh2	NA	Cdh2	cadherin 2	83501	ENSRNOG00000015602
Vcl	NA	Vcl	vinculin	305679	ENSRNOG00000010765
cellular component	plasma membrane part	GO:0044459			
C=1300;O=12;E=4.64;R=2.59;rawP=0.0018;adjP=0.0339					
Stab2	NA	Stab2	stabilin 2	282580	NULL
Cacna1a	NA	Cacna1a	calcium channel, voltage-dependent, P/Q type, alpha 1A subunit	25398	ENSRNOG00000002559
Itln1	NA	Itln1	intelectin 1 (galactofuranose binding)	498284	ENSRNOG00000004678
Cdh2	NA	Cdh2	cadherin 2	83501	ENSRNOG00000015602
Ddr2	NA	Ddr2	discoidin domain receptor tyrosine kinase 2	685781	ENSRNOG00000002881
Ephb1	NA	Ephb1	Eph receptor B1	24338	ENSRNOG00000007865
Epha4	NA	Epha4	Eph receptor A4	316539	ENSRNOG00000013213

Egfr	NA	Egfr	epidermal growth factor receptor	24329	ENSRNOG00000004332
Gria4	NA	Gria4	glutamate receptor, ionotropic, AMPA 4	29629	ENSRNOG00000006957
Ptk2	NA	Ptk2	PTK2 protein tyrosine kinase 2	25614	ENSRNOG00000007916
Nrg1	NA	Nrg1	neuregulin 1	112400	ENSRNOG00000010392
Kcnt2	NA	Kcnt2	potassium channel, subfamily T, member 2	304827	ENSRNOG00000013312

cellular component	cell periphery	GO:0071944			
C=3057;O=20;E=10.91;R=1.83;rawP=0.0032;adjP=0.0452					
Cacna1a	NA	Cacna1a	calcium channel, voltage-dependent, P/Q type, alpha 1A subunit	25398	ENSRNOG00000002559
Cdh2	NA	Cdh2	cadherin 2	83501	ENSRNOG00000015602
Ddr2	NA	Ddr2	discoidin domain receptor tyrosine kinase 2	685781	ENSRNOG00000002881
Ephb1	NA	Ephb1	Eph receptor B1	24338	ENSRNOG00000007865
Myo9b	NA	Myo9b	myosin IXb	25486	ENSRNOG00000016256
Elmo1	NA	Elmo1	engulfment and cell motility 1	361251	ENSRNOG00000018726
Gria4	NA	Gria4	glutamate receptor, ionotropic, AMPA 4	29629	ENSRNOG00000006957
Vcl	NA	Vcl	vinculin	305679	ENSRNOG00000010765
Kcnt2	NA	Kcnt2	potassium channel, subfamily T, member 2	304827	ENSRNOG00000013312
Cdh19	NA	Cdh19	cadherin 19, type 2	360835	ENSRNOG00000029841
Stab2	NA	Stab2	stabilin 2	282580	NULL
Itln1	NA	Itln1	intelectin 1 (galactofuranose binding)	498284	ENSRNOG00000004678
Ppp3cb	NA	Ppp3cb	protein phosphatase 3, catalytic subunit, beta isozyme	24675	ENSRNOG00000007757
Epha4	NA	Epha4	Eph receptor A4	316539	ENSRNOG00000013213
Cyth1	NA	Cyth1	cytohesin 1	116691	ENSRNOG00000043381
Vom2r37	NA	Vom2r37	vomeronasal 2 receptor, 37	690356	ENSRNOG00000043280
Egfr	NA	Egfr	epidermal growth factor receptor	24329	ENSRNOG00000004332
Nrg1	NA	Nrg1	neuregulin 1	112400	ENSRNOG00000010392
Ptk2	NA	Ptk2	PTK2 protein tyrosine kinase 2	25614	ENSRNOG00000007916
Rheb	NA	Rheb	Ras homolog enriched in brain	26954	NULL

cellular component	cell-cell junction	GO:0005911			
C=273;O=5;E=0.97;R=5.13;rawP=0.0028;adjP=0.0452					
Sdccag8	NA	Sdccag8	serologically defined colon cancer antigen 8	305002	ENSRNOG00000004181
Cdh2	NA	Cdh2	cadherin 2	83501	ENSRNOG00000015602
Ptk2	NA	Ptk2	PTK2 protein tyrosine kinase 2	25614	ENSRNOG00000007916
Cyth1	NA	Cyth1	cytohesin 1	116691	ENSRNOG00000043381
Vcl	NA	Vcl	vinculin	305679	ENSRNOG00000010765
